# Detecting functional connectivity disruptions in a translational pediatric traumatic brain injury porcine model using resting-state and task-based fMRI

**DOI:** 10.1038/s41598-021-91853-5

**Published:** 2021-06-11

**Authors:** Gregory Simchick, Kelly M. Scheulin, Wenwu Sun, Sydney E. Sneed, Madison M. Fagan, Savannah R. Cheek, Franklin D. West, Qun Zhao

**Affiliations:** 1 Department of Physics and Astronomy, Franklin College of Arts and Sciences, University of Georgia, 500 D.W. Brooks Drive Rm 119, Athens, GA 30602 USA; 2 Regenerative Bioscience Center, University of Georgia, 425 River Road Rm 316, Athens, GA 30602 USA; 3 Biomedical and Health Sciences Institute, Neuroscience Program, University of Georgia, Athens, GA USA; 4 Department of Animal and Dairy Science, College of Agricultural and Environmental Sciences, University of Georgia, Athens, GA USA

**Keywords:** Motor cortex, Computational neuroscience, Neural circuits, Sensorimotor processing, Trauma, Functional magnetic resonance imaging, Magnetic resonance imaging

## Abstract

Functional magnetic resonance imaging (fMRI) has significant potential to evaluate changes in brain network activity after traumatic brain injury (TBI) and enable early prognosis of potential functional (e.g., motor, cognitive, behavior) deficits. In this study, resting-state and task-based fMRI (rs- and tb-fMRI) were utilized to examine network changes in a pediatric porcine TBI model that has increased predictive potential in the development of novel therapies. rs- and tb-fMRI were performed one day post-TBI in piglets. Activation maps were generated using group independent component analysis (ICA) and sparse dictionary learning (sDL). Activation maps were compared to pig reference functional connectivity atlases and evaluated using Pearson spatial correlation coefficients and mean ratios. Nonparametric permutation analyses were used to determine significantly different activation areas between the TBI and healthy control groups. Significantly lower Pearson values and mean ratios were observed in the visual, executive control, and sensorimotor networks for TBI piglets compared to controls. Significant differences were also observed within several specific individual anatomical structures within each network. In conclusion, both rs- and tb-fMRI demonstrate the ability to detect functional connectivity disruptions in a translational TBI piglet model, and these disruptions can be traced to specific affected anatomical structures.

## Introduction

Traumatic brain injury (TBI) is a worldwide socio-economic problem that is regularly referred to as a “silent epidemic” with 2.87 million people annually suffering from a TBI in the United States alone^1^. The largest affected population are children under 14 years of age, which is an early developmental period with increased vulnerability to injury^2,3^. Pediatric TBI can lead to life-long effects on brain structure and function and cause significant cognitive, behavioral, and motor function impairments^4^. Due to the substantial effects of TBI, there have been significant efforts to develop better magnetic resonance imaging (MRI) approaches to detect and characterize changes in neural network activity to predict functional outcomes and to generate animal models that are more representative of human TBI responses. These efforts have resulted in highly sensitive resting-state and task-based functional MRI (rs- and tb-fMRI) approaches and a porcine TBI model that has similar brain anatomy, physiology, and TBI pathophysiology to humans^3,5–9^. To date, rs- and tb-fMRI has not been performed in the TBI piglet model; raising questions as to how functional network connectivity changes in the piglet brain after TBI.

fMRI is a powerful tool that can be used to characterize injury severity and recovery in specific functional networks with known functional tasks (e.g. cognition) and can be used to predict potential deficits (e.g. learning, memory, motor) with precision in TBI patients^10–13^. Rs- and tb-fMRI have both been shown to have substantial clinical and pre-clinical potential to assess TBI injury and recovery^5–7^. On one hand, rs-fMRI can easily be conducted with minors, elderly, or mentally challenged populations^14^, and the intrinsic connectivity of neural networks mapped by rs-fMRI may be more heritable than task-elicited activity^15^. On the other hand, network mapping using tb-fMRI are found to be similar to those mapped by rs-fMRI, and tb-fMRI has been used for studies of intrinsic connectivity when rs-fMRI data is not available^16^. Furthermore, tb-fMRI has been found to underline individual differences in network dynamics^17,18^. Recently, a general functional connectivity approach was proposed for leveraging shared features across rs- and tb-fMRI^19^, and by doing so, this method was found to improve the reliability of intrinsic connectivity estimates and identify meaningful correlates of individual differences in behavior. Therefore, a combined rs- and tb-fMRI approach is important in monitoring system-level changes within and between brain functional networks after TBI. fMRI also enables assessment of critical network changes, including (1) changes in activation timing, which can enable identification of specifically affected subnetworks, (2) detection of the type of functional alteration (e.g. hypoactivation vs hyperactivation), and (3) potential reorganization of network signaling pathways. Predicting changes in cognition, behavior, and similar long-term outcomes in TBI patients has proven to be imprecise using standard clinical assessments^20^. However, fMRI has shown promise as a prognostic modality, as it has been used to successfully predict patient cognitive deficits even six months after injury^20^. Our research team has recently identified multiple resting-state networks (RSNs) in the piglet brain, including the visual (VIS), executive control (EX), sensorimotor (SM), cerebellar (CERE), and default mode (DMN) networks^9^. However, it has yet to be examined how these RSNs change in response to TBI. Additionally, in humans, the salient (SAL) and basal ganglia (BAS) networks are important in conscious task-positive activities, notably motor control^21,22^, and these networks have yet to be studied in the pig. Since common deficits in TBI patients include motor and behavioral abnormalities, the evaluation of the SAL and BAS networks may provide insight into functional connectivity responses and disruptions caused by TBI.

Our research group has developed and characterized a controlled cortical impact (CCI) piglet TBI model with cognitive and motor function deficits^23–25^. The pig was selected as a model species as the pig brain has more comparable anatomy and physiology to the human brain than the rodent, the most commonly used TBI animal model. Both pig and human brains are gyrencephalic, unlike the lissencephalic rodent brain, which is directly correlated with brain connectivity and complexity^26–28^. In the gyrencephalic brain, TBI mechanical stress is focused near the base of the sulci and is more heterogeneously distributed relative to the lissencephalic brain^29^. In addition, brain size plays a crucial role in TBI. Smaller rodent brains can tolerate greater angular acceleration forces opposed to larger pig and human brains. In the CCI TBI piglet model, deficits associated with the SM network have been observed, including gait changes such as increased stance time, decreased velocity, percent of two limb support, and proprioception^25,30,31^. These changes are commonly observed in human TBI patients with SM damage and are due to a loss of coordination, lack of bilateral arm/leg coupling, and reduced agility and balance^32^. Additionally, TBI piglets displayed impairments in exploratory behaviors and spatial memory formation, which are associated with the EX network^25^. The similarities between pig and human brain anatomy, physiology, brain network activity, cognition, and motor function responses to TBI make the pig an attractive translational model to study functional network changes due to TBI.

In this study, both rs- and tb-fMRI are employed to examine the functional connectivity disruptions caused by TBI using a porcine CCI model. A reference pig functional connectivity atlas was constructed for seven different networks using a previously published pig anatomical atlas^33^. Functional connectivity activation maps for each individual piglet were generated using two methods: a group independent component analysis (ICA) with back reconstruction method and a group sparse dictionary learning (sDL) with dual regression method. The activation maps generated from each method were compared to each reference atlas using Pearson spatial correlation coefficients and mean ratios to evaluate differences between healthy control and TBI groups. To further locate and evaluate significantly different voxels between the TBI and control groups, a nonparametric permutation test was performed using the activation maps generated by the sDL analysis.

## Results

### Reference atlas analysis

#### rs-fMRI detects differences in VIS and EX networks

**Figure 1:**
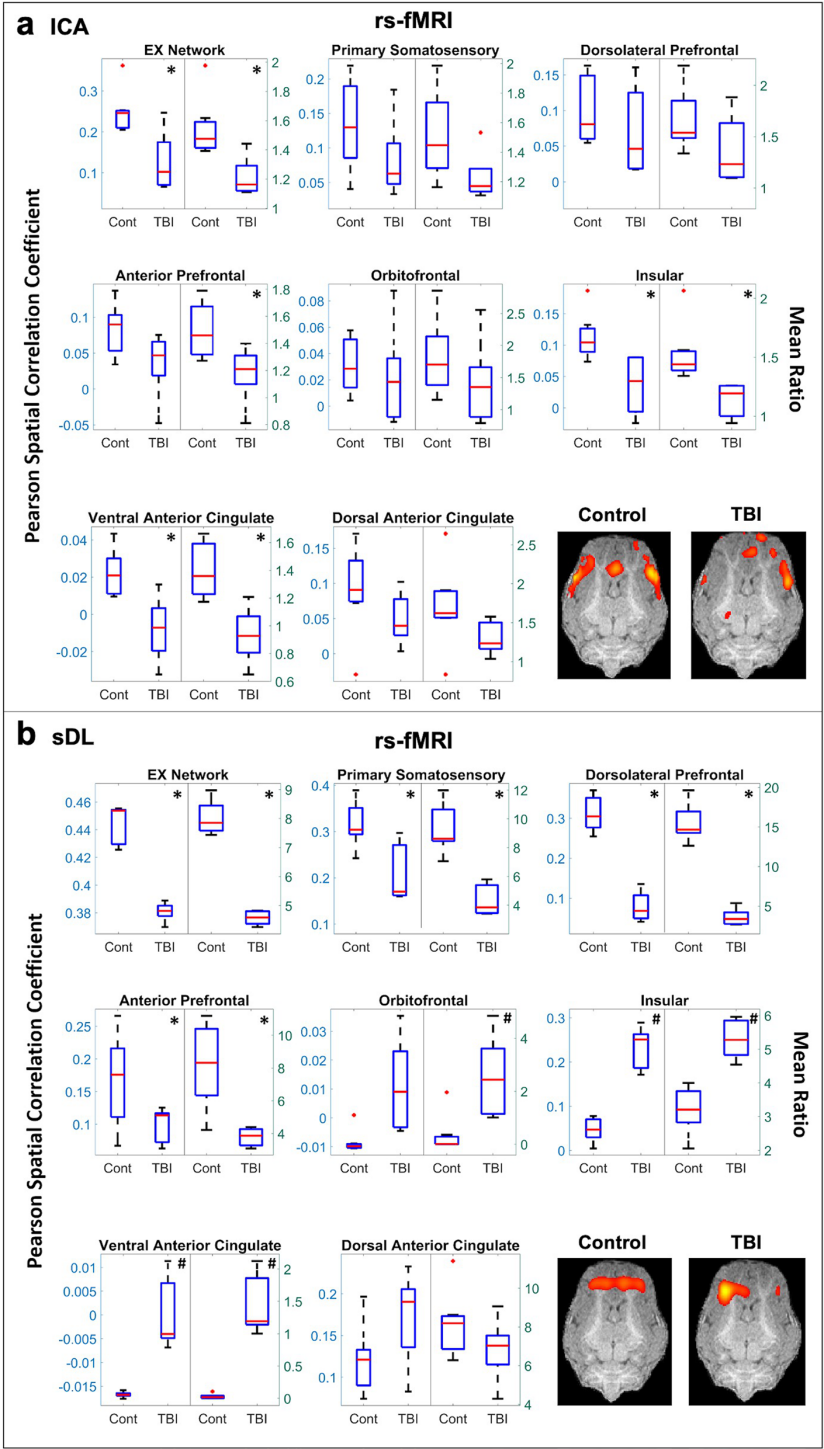
Resting-state (rs-) analysis of the executive control (EX) network. Boxplots of Pearson spatial correlation coefficients (left) and mean ratios (right) obtained from the rs- analysis for the EX network and its individual anatomical regions obtained using independent component analysis (ICA; **a**) and sparse dictionary learning (sDL; **b**). The red line denotes the median, the blue box denotes the interquartile range, and the lower and upper whiskers denote the min and max, respectively. An asterisk (*) or pound (#) in the upper right-hand corner indicates significant decrease or increase (p < 0.05), respectively, for the TBI group in comparison to the control group. Representative coronal plane activation maps overlaid on the template pigs’ T1-weighted anatomical image are also displayed. Representative maps in the axial and sagittal plane are provided in the Figures S4 and S5.

When examining the activation maps obtained from the rs-fMRI data, significant decreases (p < 0.05) in the Pearson spatial correlation coefficients and mean ratios for the TBI group in comparison to the control group for the EX network were observed using both ICA and sDL (Table 1a and Fig. 1). Significant decreases in individual structures comprising the EX network (the primary somatosensory, dorsolateral prefrontal, and anterior prefrontal cortices) were observed using sDL (Fig. 1b), and although not significant, similar decreasing patterns were observed for these structures using ICA (Fig. 1a). However, contradictory decreasing and increasing significant differences were observed in the insular and ventral anterior cingulate cortices for ICA and sDL, respectively. No statistical differences were observed for the orbitofrontal or dorsal anterior cingulate cortices using either method.

**Table 1:** P-Values Comparing Control vs TBI Group.

Networks and anatomical regions	ICA			sDL		
	Sig	Pearson	Mean Ratio	Sig	Pearson	Mean Ratio
**a. rs-fMRI**						
**Executive control**^**$**^	*	0.02*	< 0.01*	*	< 0.01*	< 0.01*
EX1: primary somatosensory cortex^**$**^	–	0.19	0.07	*	0.02*	< 0.01*
EX2: dorsolateral prefrontal cortex^**$**^	–	0.42	0.17	*	< 0.01*	< 0.01*
EX3: anterior prefrontal cortex^**$**^	–	0.10	0.03*	*	0.04*	< 0.01*
EX4: orbitofrontal cortex	–	0.66	0.31	–	0.06	0.03^#^
EX5: insular cortex	*	0.02*	< 0.01*	^#^	< 0.01^#^	< 0.01^#^
EX6: ventral anterior cingulate cortex	*	0.02*	< 0.01*	^#^	< 0.01^#^	< 0.01^#^
EX7: dorsal anterior cingulate cortex	–	0.18	0.11	–	0.14	0.20
**Sensorimotor**	–	0.92	0.67	–	0.46	0.31
SM1: primary motor cortex	–	0.06	0.11	–	0.75	0.54
SM2: somatosensory associative cortex^**$**^	–	0.02^#^	0.07	–	0.17	0.16
SM3: premotor cortex^**$**^	*	< 0.01*	< 0.01*	–	0.29	0.42
**Cerebellar**	–	0.15	0.09	–	0.09	0.77
**Visual**^**$**^	–	0.11	0.10	*	< 0.01*	0.02*
VIS1: primary visual cortex^**$**^	–	0.54	0.20	*	0.01*	0.01*
VIS2: secondary visual cortex^**$**^	–	0.06	0.06	–	0.22	0.30
VIS3: associative visual cortex^**$**^	–	0.10	0.10	*	0.02*	0.02*
**b. tb**-**fMRI**						
**Executive Control (tactile stimulus)** ^$^	*	< 0.01*	0.03*	–	0.04*	0.07
EX1: Primary Somatosensory Cortex^$^	*	< 0.01*	0.03*	*	0.02*	< 0.01*
EX2: dorsolateral prefrontal cortex	–	0.23	0.03*	–	0.57	0.91
EX3: anterior prefrontal cortex	–	0.13	0.41	–	0.46	0.43
EX4: orbitofrontal cortex^$^	–	0.15	0.12	–	0.27	0.25
EX5: Insular Cortex^$^	*	< 0.01*	0.04*	–	0.06	0.05
EX6: ventral anterior cingulate cortex^$^	^#^	< 0.01^#^	< 0.01^#^	–	0.12	0.08
EX7: dorsal anterior cingulate cortex	–	0.13	0.32	–	0.37	0.68
**Sensorimotor (tactile stimulus)** ^$^	*	< 0.01*	< 0.01*	–	0.12	0.15
SM1: primary motor cortex^$^	*	< 0.01*	< 0.01*	–	0.24	0.24
SM2: somatosensory associative cortex^$^	*	< 0.01*	< 0.01*	–	0.29	0.29
SM3: premotor cortex^$^	–	0.51	0.12	–	0.10	0.12
**Cerebellar (tactile stimulus)** ^$^	–	0.10	0.31	–	0.13	0.21
**Visual (visual stimulus)** ^$^	–	0.66	0.41	–	0.92	0.91
VIS1: primary visual cortex^$^	–	0.80	0.48	–	0.99	0.93
VIS2: secondary visual cortex^$^	–	0.46	0.34	–	0.80	0.94
VIS3: associative visual cortex^$^	–	0.65	0.98	–	0.83	0.85

P-values comparing the Pearson spatial correlation coefficients and mean ratios obtained from the resting-state (rs-) fMRI and task-based (tb-) fMRI analysis for the control group and the TBI group for each network and each individual anatomical region. For each metric, the groups were considered significantly different if p < 0.05 (denoted by * for a significant decrease in the TBI group in comparison to the control group and ^#^ for a significant increase). However, a network or anatomical region was only considered significantly different if the p-values for both the Pearson values and mean ratios were below 0.05 (‘Sig’ columns). Networks and regions that showed consistent trends across ICA and sDL are denoted by ^$^.

**Figure 2:**
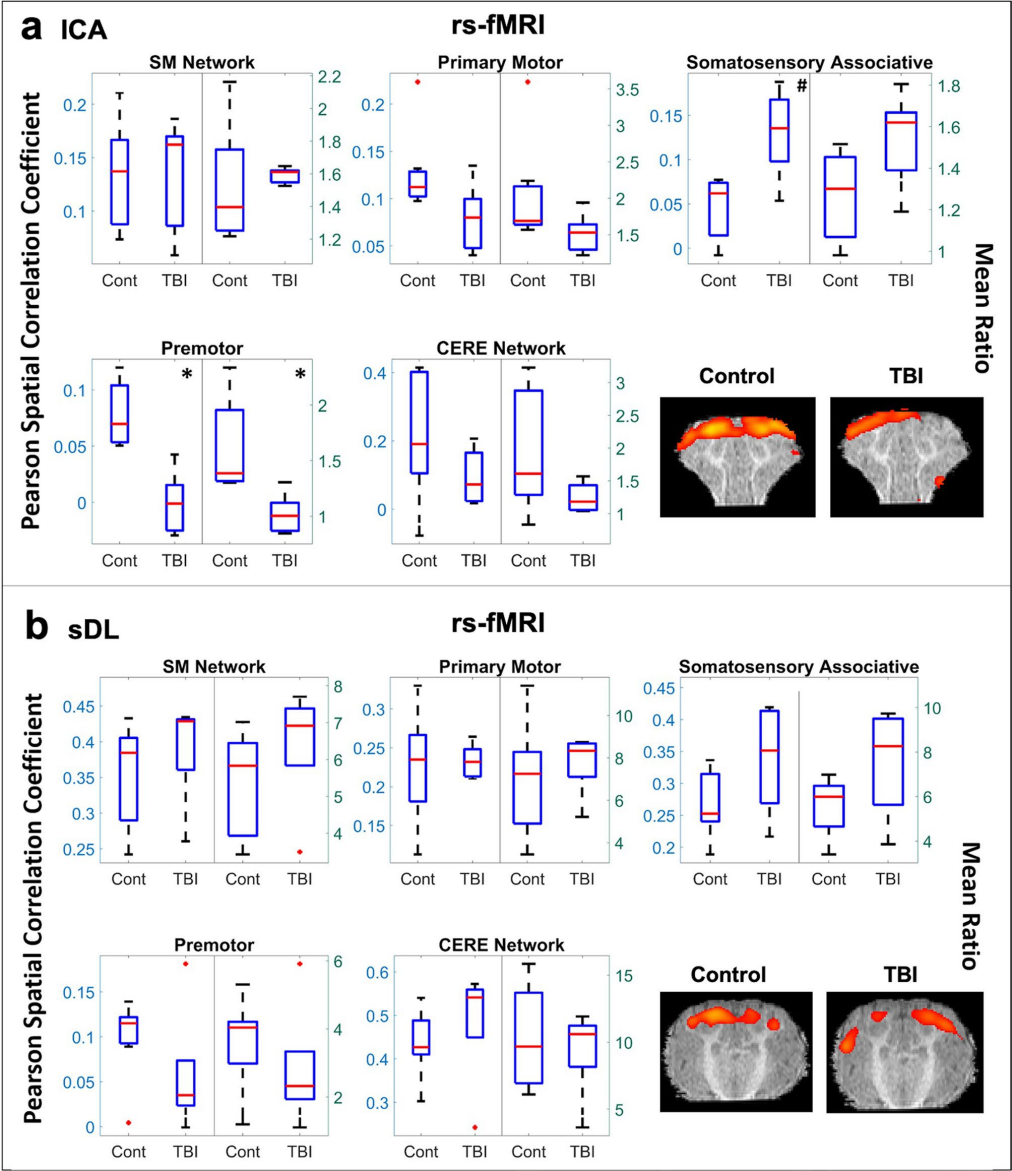
Resting-state (rs-) analysis of the sensorimotor (SM) and cerebellar (CERE) networks. Boxplots of Pearson spatial correlation coefficients (left) and mean ratios (right) obtained from the rs- analysis for the SM network and its individual anatomical regions, as well as the CERE network, obtained using independent component analysis (ICA; **a**) and sparse dictionary learning (sDL; **b**). The red line denotes the median, the blue box denotes the interquartile range, and the lower and upper whiskers denote the min and max, respectively. An asterisk (*) or pound (#) in the upper right-hand corner indicates significant decrease or increase (p < 0.05), respectively, for the TBI group in comparison to the control group. Representative coronal plane activation maps overlaid on the template pigs’ T1-weighted anatomical image are also displayed. Representative maps in the axial and sagittal plane are provided in the Figures S4 and S5.

No statistical differences were observed in the TBI group compared to the control group for the SM or CERE networks (Table 1a and Fig. 2). However, within the SM network, a significant decrease in the premotor cortex was observed using ICA (Fig. 2a), and a similar decrease was observed using sDL (Fig. 2b). A consistent increasing trend for the somatosensory associative cortex was also observed, while no statistical difference was observed for the primary motor cortex.

**Figure 3:**
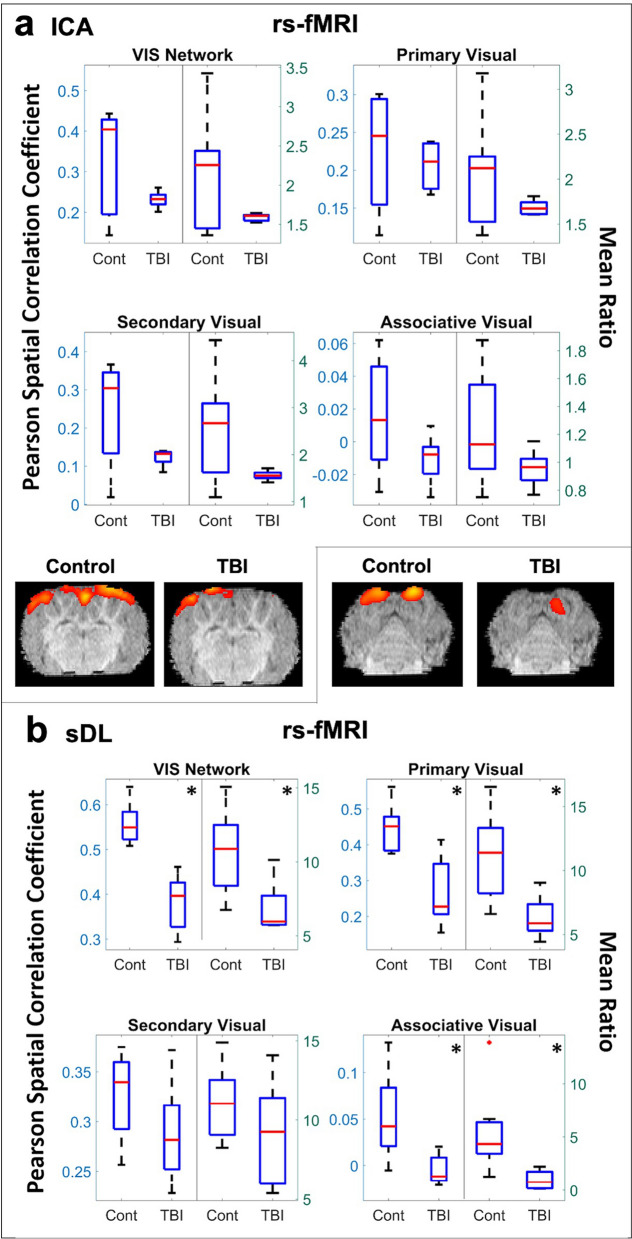
Resting-state (rs-) analysis of the visual (VIS) network. Boxplots of Pearson spatial correlation coefficients (left) and mean ratios (right) obtained from rs- analysis for the VIS network and its individual anatomical regions obtained using independent component analysis (ICA; **a**) and sparse dictionary learning (sDL; **b**). The red line denotes the median, the blue box denotes the interquartile range, and the lower and upper whiskers denote the min and max, respectively. An asterisk (*) or pound (#) in the upper right-hand corner indicates significant decrease or increase (p < 0.05), respectively, for the TBI group in comparison to the control group. Representative axial plane activation maps overlaid on the template pigs’ T1-weighted anatomical image are also displayed. Representative maps in the coronal and sagittal plane are provided in the Figures S4 and S5.

For the VIS network, a significant decrease was observed using sDL (Table 1a and Fig. 3b), and although a significant difference was not observed when using ICA to detect functional networks, a similar decreasing pattern was observed (Fig. 3a). When examining the individual structures comprising the VIS network, the primary and associative visual cortices displayed the same significant decreases as the whole network for sDL, and ICA displayed similar decreasing patterns. No statistical difference was observed for the secondary visual cortex using either sDL or ICA. However, both methods consistently showed decreased activation compared to controls for this anatomical structure.

No statistical differences were observed in the TBI group in comparison to the control group for the DMN, SAL, or BAS networks using either method (Table S1 and Figures S1-3 of the Supplementary Material). Overall, relatively low Pearson values and mean ratios were observed for these networks, even for the control group. Therefore, it’s possible that these networks weren’t able to be properly detected.

In Figs. 1, 2, 3, single plane representative images of two-dimensional activation maps are also presented for the control and TBI group. However, it is important to note that these images only show a cross-section of the brain, whereas the Pearson values and mean ratios are calculated based on the entire three-dimensional volume of the brain. Additional cross-sectional images in all three planes for each network and method for the rs-fMRI analysis are provided in Figures S4a,b and S5a,b.

#### tb-fMRI detects differences in EX and SM networks

**Figure 4:**
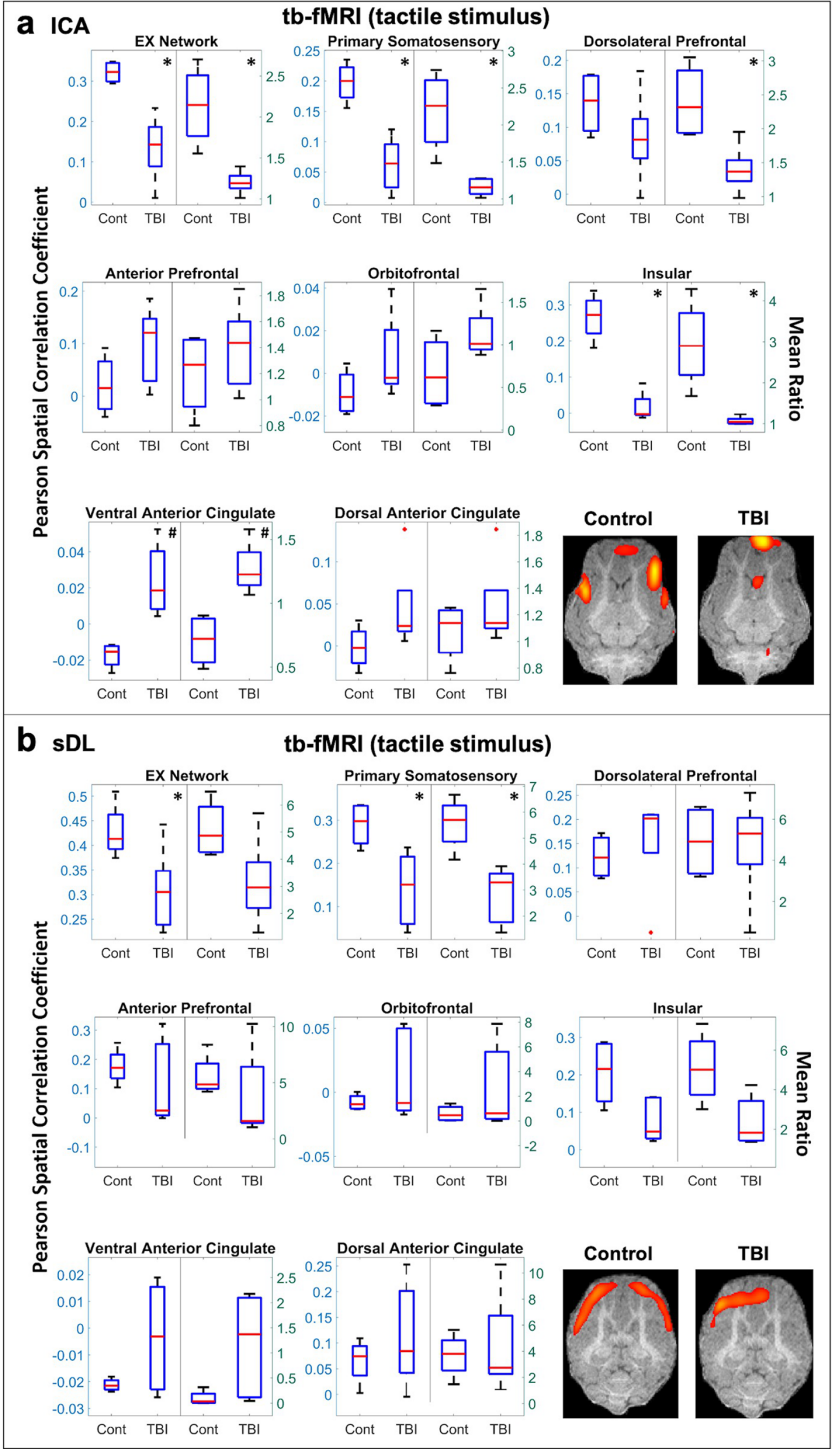
Task-based (tb-) tactile stimulus analysis of the executive control (EX) network. Boxplots of Pearson spatial correlation coefficients (left) and mean ratios (right) obtained from the tb- analysis for the EX network and its individual anatomical regions obtained using independent component analysis (ICA; **a**) and sparse dictionary learning (sDL; **b**). The red line denotes the median, the blue box denotes the interquartile range, and the lower and upper whiskers denote the min and max, respectively. An asterisk (*) or pound (#) in the upper right-hand corner indicates significant decrease or increase (p < 0.05), respectively, for the TBI group in comparison to the control group. Representative coronal plane activation maps overlaid on the template pigs’ T1-weighted anatomical image are also displayed. Representative maps in the axial and sagittal plane are provided in the Figures S4 and S5.

When examining the activation maps obtained from the tb-fMRI tactile stimulus data, a significant decrease in the EX network using ICA was observed (Table 1b and Fig. 4a), and a similar decreasing trend was observed using sDL (Fig. 4b). Within the EX network, the primary somatosensory and insular cortices displayed similar significant decreases using ICA. sDL also found a significant decrease in the primary somatosensory cortex and displayed a similar decreasing pattern in the insular cortex. An increasing trend in the ventral anterior cingulate cortex was observed using ICA and supported by a similar increasing pattern observed using sDL. No statistical differences were observed for the dorsolateral prefrontal, anterior prefrontal, orbitofrontal, or dorsal anterior cingulate cortices using either method.

**Figure 5:**
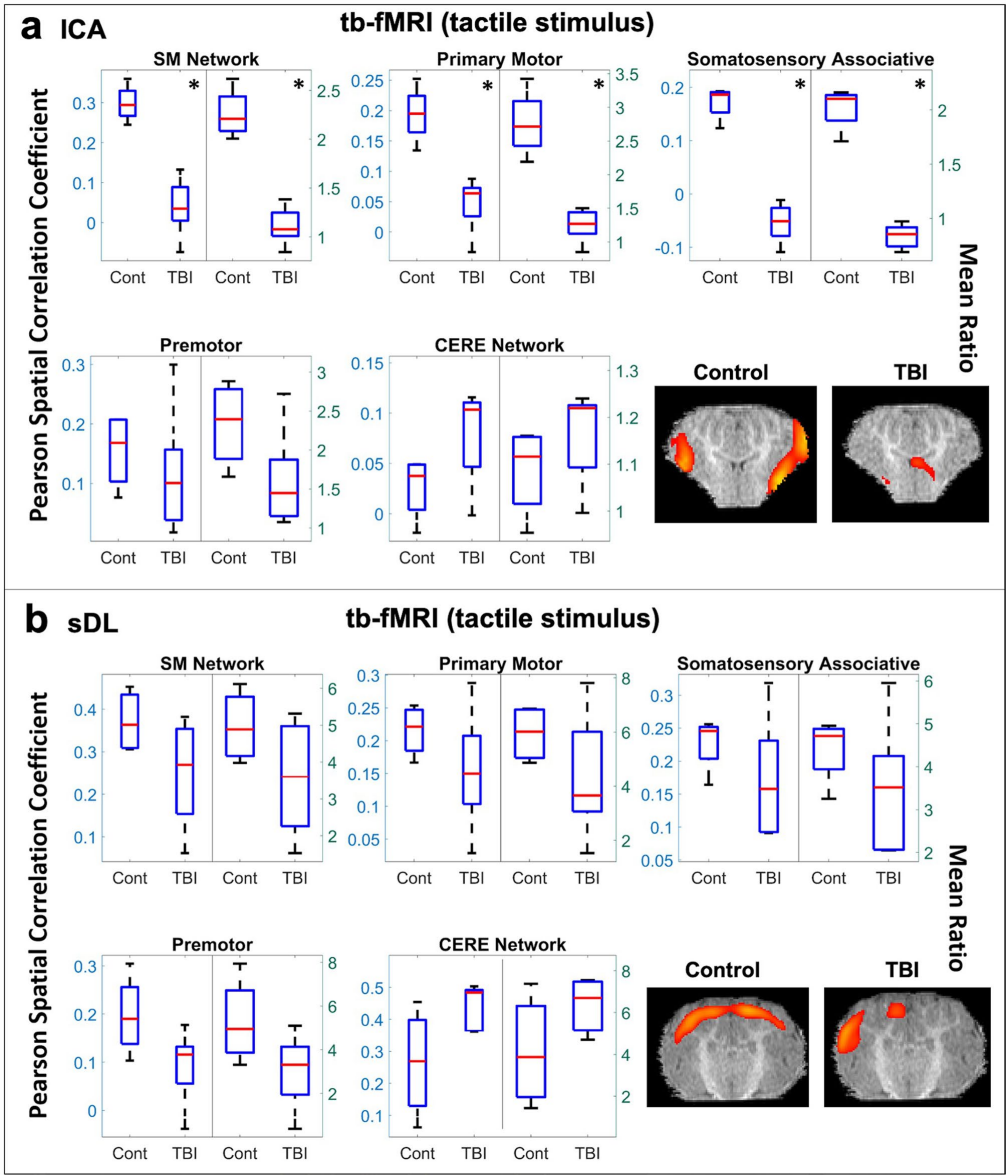
Task-based (tb-) tactile stimulus analysis of the sensorimotor (SM) and cerebellar (CERE) networks. Boxplots of Pearson spatial correlation coefficients (left) and mean ratios (right) obtained from the tb- analysis for the SM network and its individual anatomical regions, as well as the CERE network, obtained using independent component analysis (ICA; **a**) and sparse dictionary learning (sDL; **b**). The red line denotes the median, the blue box denotes the interquartile range, and the lower and upper whiskers denote the min and max, respectively. An asterisk (*) or pound (#) in the upper right-hand corner indicates significant decrease or increase (p < 0.05), respectively, for the TBI group in comparison to the control group. Representative coronal plane activation maps overlaid on the template pigs’ T1-weighted anatomical image are also displayed. Representative maps in the axial and sagittal plane are provided in the Figures S4 and S5.

A significant decrease in the TBI group in comparison to the control group was also observed for the SM network using ICA to analyze the tb-fMRI tactical stimulus data (Table 1b and Fig. 5a), and the sDL results showed a similar decreasing pattern (Fig. 5b). Within the SM network, the primary motor and somatosensory associative cortices displayed significant decreases using ICA, and sDL displayed similar decreasing patterns. Although not significant, a consistent decreasing pattern was also observed for the premotor cortex for both methods, and a consistent increase was observed in TBI animals in the CERE network (Table 1b and Fig. 5a,b).

**Figure 6:**
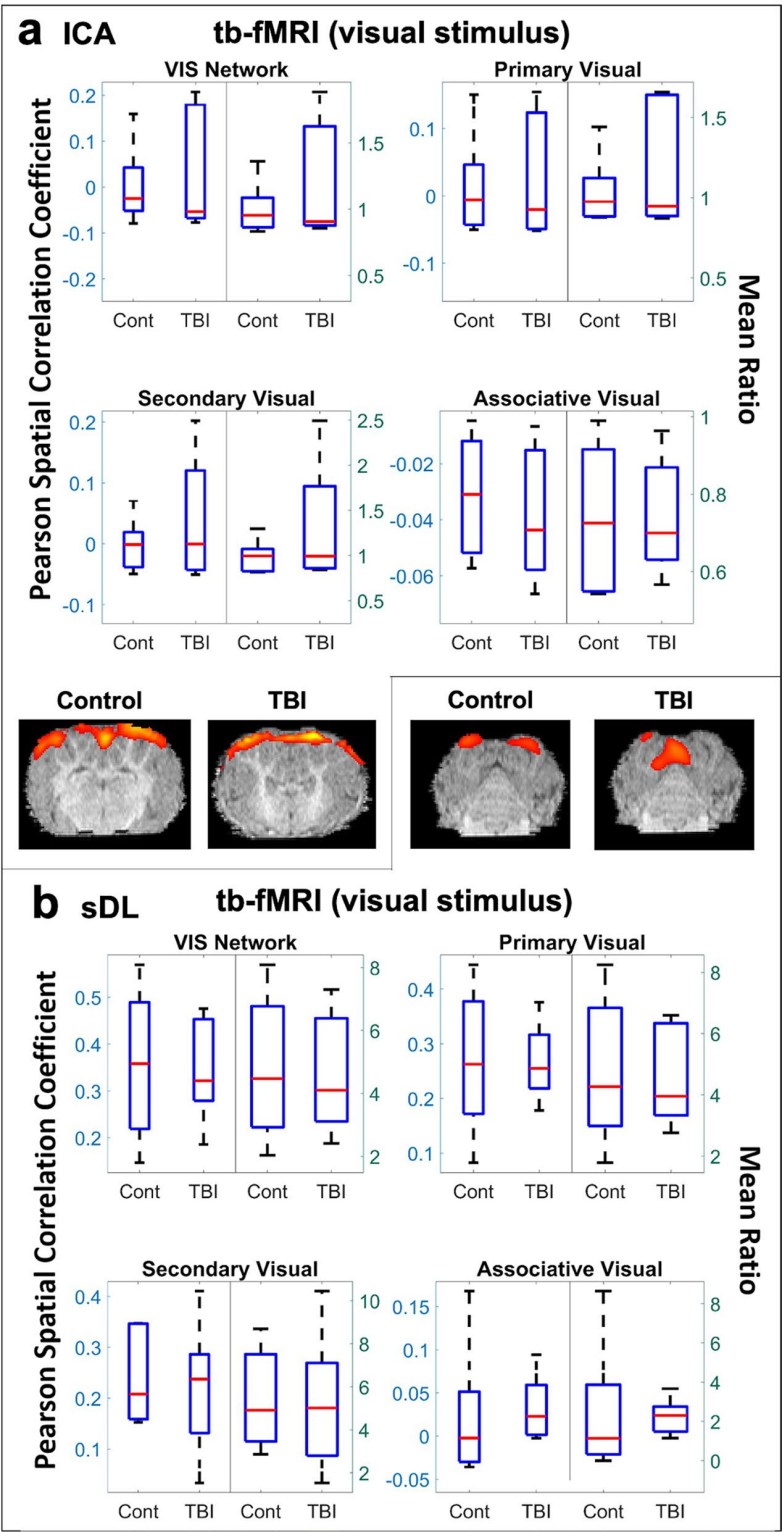
Task-based (tb-) visual stimulus analysis of the visual (VIS) network. Boxplots of Pearson spatial correlation coefficients (left) and mean ratios (right) obtained from the tb- analysis for the VIS network and its individual anatomical regions obtained using independent component analysis (ICA; **a**) and sparse dictionary learning (sDL; **b**). The red line denotes the median, the blue box denotes the interquartile range, and the lower and upper whiskers denote the min and max, respectively. An asterisk (*) or pound (#) in the upper right-hand corner indicates significant decrease or increase (p < 0.05), respectively, for the TBI group in comparison to the control group. Representative axial plane activation maps overlaid on the template pigs’ T1-weighted anatomical image are also displayed. Representative maps in the coronal and sagittal plane are provided in the Figures S4 and S5.

When examining the activation maps obtained from the tb-fMRI visual stimulus data, no statistical difference in the Pearson spatial correlation coefficients or mean ratios for the TBI group in comparison to the control group were observed for the VIS network or any of its individual anatomical structures using ICA or sDL (Table 1b and Fig. 6).

Single plane representative images of two-dimensional activation maps are also presented for the control and TBI group in Figs. 4, 5, 6. Again, it is important to note that these images only show a cross-section of the brain, whereas the Pearson values and mean ratios are calculated based on the entire three-dimensional volume of the brain. Additional cross-sectional images in all three planes for each network and method for the tb-fMRI analysis are provided in Figures S4c,d and S5c,d.

### Permutation analysis

#### Permutation analysis of rs-fMRI detects disruptions in SM and CERE networks

**Figure 7:**
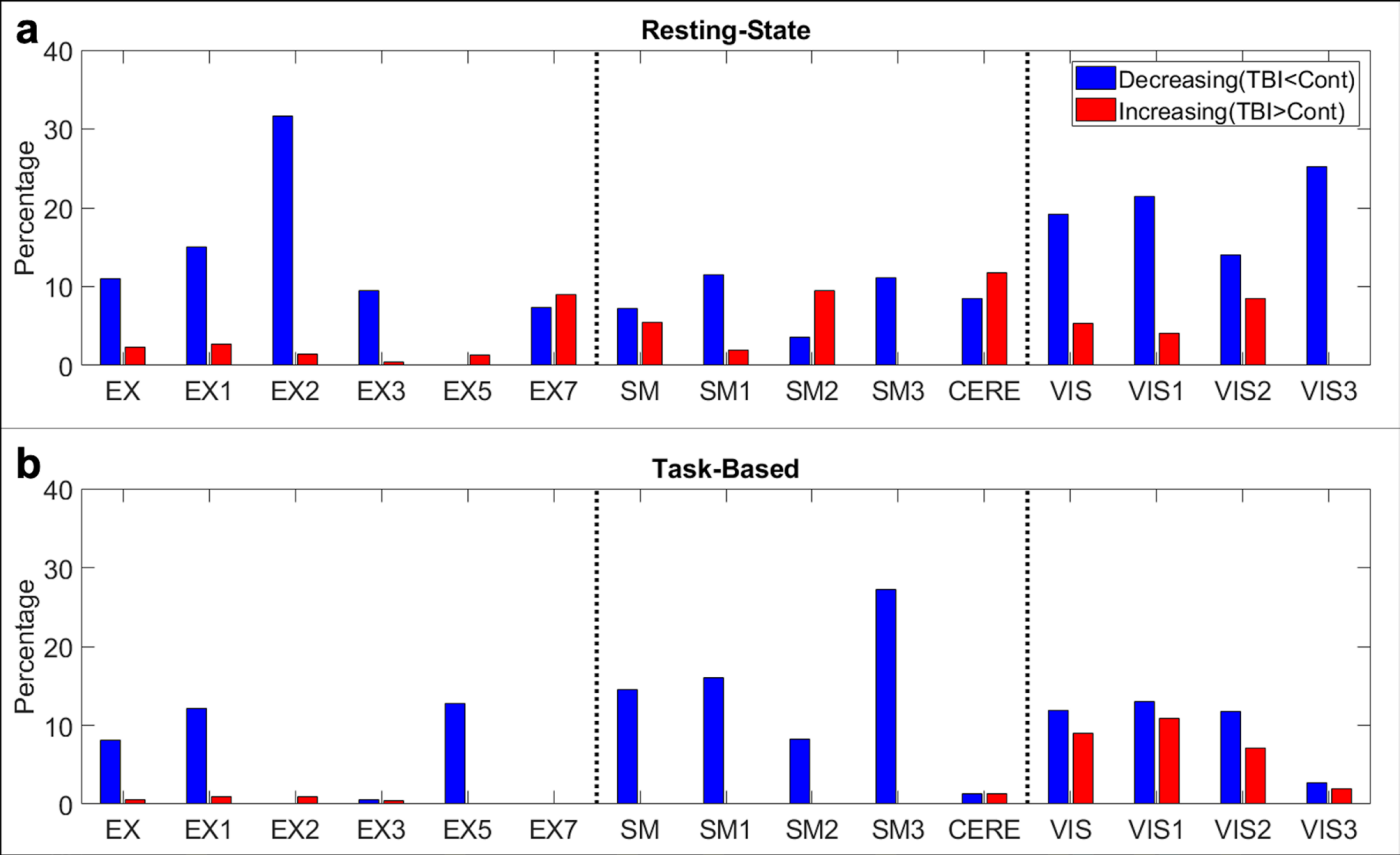
Permutation analysis percentages of significantly different voxels. Bar plots of the percentages of significantly decreasing (blue, TBI < Control) and increasing (red, TBI > Control) voxels contained within each network and each individual anatomical region comprising each network for the resting-state (**a**) and task-based (**b**) fMRI permutation analysis. Abbreviations: visual (VIS), executive control (EX), sensorimotor (SM), and cerebellar (CERE). Regions EX4 and EX6, corresponding to the orbitofrontal and ventral anterior cingulate cortices, are not shown, as they did not contain any significantly different voxels for either analysis. For identification of other regions, refer to Table 1.

Since sDL outperformed ICA at detecting the examined networks in the control group, as observed by the higher measured Pearson values and mean ratios (Figs. 1, 2, 3, 4, 5, 6), permutation analysis was performed using the activation maps generated using the sDL methodology. The percentages of voxels that contained significantly decreasing and increasing activation values for the TBI group in comparison to the control group for each network and individual anatomical structure for the rs- and tb-fMRI data are shown in Fig. 7.

When examining the activation maps obtained from the rs-fMRI data using permutation analysis, the EX network, as well as all comprising individual anatomical structures except the insular (EX5) and dorsal anterior cingulate (EX7) cortices, contained higher percentages of significantly decreasing voxels than increasing voxels (> 9.0% decreasing in comparison to < 3.0% increasing; Fig. 7a). This agrees with the trends observed in rs-fMRI reference atlas analysis (Figs. 1). However, it was observed that the insular cortex only contained a small percentage of significantly increasing voxels (1.3%), and the dorsal anterior cingulate cortex contained relatively similar percentages of significantly decreasing and increasing voxels (7.4% and 9.0%, respectively).

The SM network as a whole also contained relatively similar percentages of significantly decreasing and significantly increasing voxels (7.2% and 5.5%, respectively), with the primary motor (SM1) and premotor (SM3) cortices showing relatively higher percentages of significantly decreasing voxels (11.5% and 11.1% decreasing in comparison to 1.9% and 0.0% increasing, respectively) and the somatosensory associative cortex (SM2) showing a relatively higher percentage of significantly increasing voxels (9.5% increasing in comparison to 3.6% decreasing; Fig. 7a). The CERE network contained a slightly higher percentage of significantly increasing voxels (11.7%) than significantly decreasing voxels (8.5%). Except for the primary motor cortex, these observations also agree with the trends observed in rs-fMRI reference atlas analysis (Fig. 2). Although no statistical differences were observed in the Pearson values and mean ratios when examining the SM and CERE networks, relatively large percentages of voxels within these networks are observed to be significantly different using permutation analysis.

**Figure 8:**
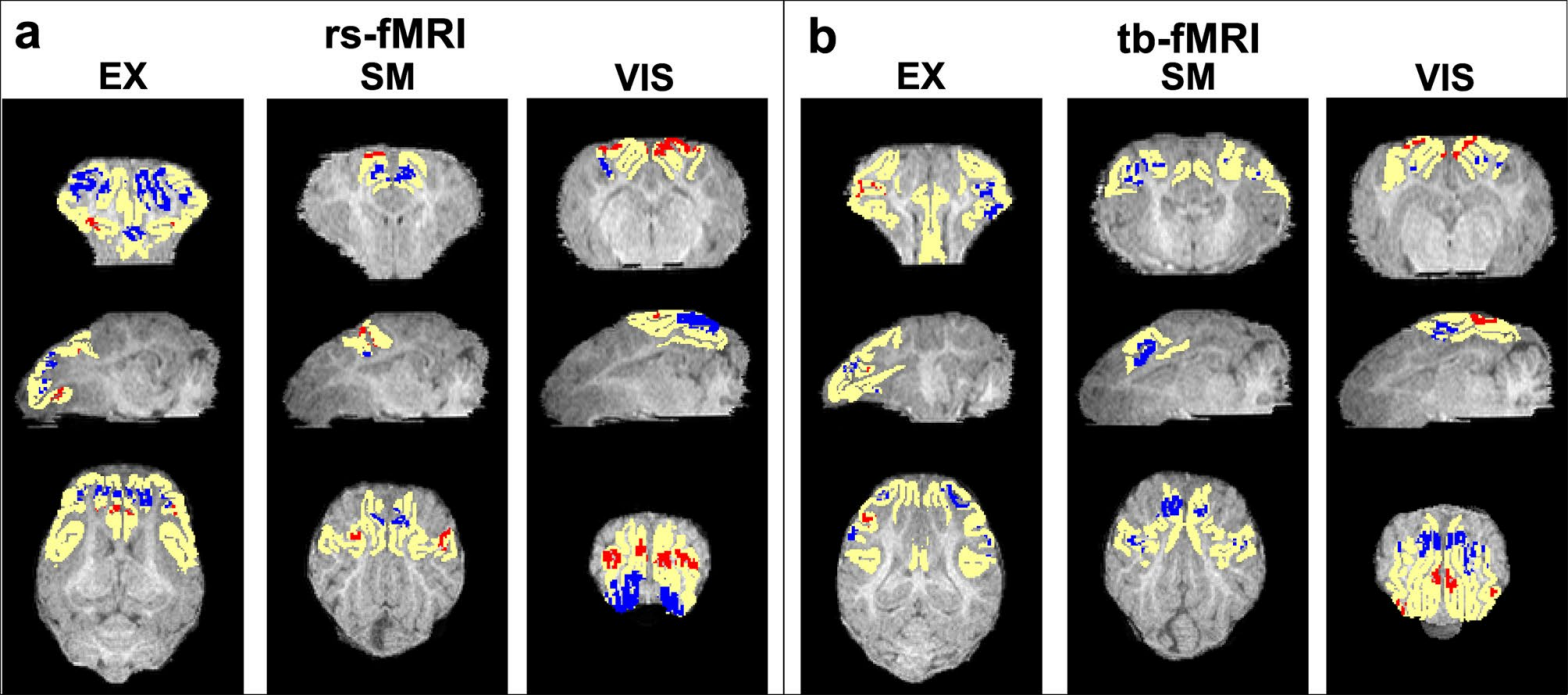
Permutation maps generated from sDL analysis. Representative images of two-dimensional permutation maps from the resting-state (rs-; **a**) and task- based (tb-; **b**) fMRI analysis for the visual (VIS), executive control (EX), and sensorimotor (SM) networks and their corresponding reference atlases (yellow) overlaid on the template pigs’ T1-weighted anatomical image. The blue indicates voxels with significantly decreased activation values of the TBI group in comparison to the control group, and red indicates significantly increased activation values.

The VIS network, as well as all of its comprising individual anatomical structures (primary, secondary, and associative visual cortices), also contained relatively higher percentages of significantly decreasing voxels than increasing voxels (19.2%, 21.5%, 14.0% and 25.2% decreasing in comparison to 5.3%, 4.1%, 8.5% and 0.0% increasing, respectively; Fig. 7a). Figure 8a displays representative images of two-dimensional permutation maps generated for the rs-fMRI analysis for the VIS, EX, and SM networks and their corresponding reference atlases overlaid on the template pigs’ T1-weighted anatomical images.

#### Permutation analysis of tb-fMRI detects differences in the VIS network

When using permutation analysis to examine the activation maps obtained from the tb-fMRI tactile stimulus data, both the EX and SM networks contained relatively higher percentages of significantly decreasing voxels than increasing voxels (8.2% and 14.6% decreasing in comparison to 0.6% and 0.0% increasing, respectively; Fig. 7b). In the EX network, the primary somatosensory (EX1) and insular (EX5) cortices contained the largest percentages of significantly decreasing voxels (12.2% and 12.8%, respectively), while all other structures contained relatively small percentages of significantly different voxels (< 1.0%). In the SM network, all three structures contained relatively high percentages of significantly decreasing voxels (> 8.0%). These observations agree with the trends observed in the pig reference atlas analysis (Figs. 4, 5). The CERE network, which showed a slight increasing trend in the pig reference analysis (Fig. 5), contained the same small percentage of significantly decreasing and significantly increasing voxels (1.3% each) for the permutation analysis (Fig. 7b).

When using permutation analysis to examine the activation maps obtained from the tb-fMRI visual stimulus data, the VIS network, including the primary (VIS1) and secondary visual (VIS2) cortices, contained higher, although relatively similar, percentages of significantly decreasing voxels than significantly increasing voxels (11.9% in VIS, 13.0% in VIS1, and 11.8% in VIS2 decreasing in comparison to 8.9% in VIS, 10.9% in VIS1, and 7.1% in VIS2 increasing; Fig. 7b). The associative visual cortex (VIS3) contained similarly small percentages of significantly decreasing and significantly increasing voxels (2.7% and 2.0%, respectively). Although no statistical difference was observed in the Pearson values and mean ratios when examining the VIS network using the pig reference atlas analysis (Fig. 6), relatively large percentages of voxels within this network and its individual anatomical structures were observed to be significantly different using permutation analysis.

Figure 8b displays representative images of two-dimensional permutation maps generated for the tb-fMRI analysis for the VIS, EX, and SM networks and their corresponding reference atlases overlaid on the template pigs’ T1-weighted anatomical image.

## Discussion

In this study, functional networks were examined using rs- and tb-fMRI in a pediatric porcine model of TBI and healthy age-matched controls to determine changes in functional connectivity after insult. Capitalizing on the advantages of both ICA and sDL, functional connectivity disruptions in the piglet TBI brain were consistently detected in the EX and VIS networks using rs-fMRI, as well as the EX and SM networks using tb-fMRI (Table 1, and Figs. 1, 3, 4, and 5). Pearson values and mean ratios showed significant reductions in these networks in the TBI group relative to the control group. The relatively high percentages of significantly decreasing voxels within these networks determined by the permutation analysis also provides additional evidence of these disruptions (Fig. 7). Although significantly different Pearson values and mean ratios were not observed for the SM and CERE networks using rs-fMRI (Fig. 2) or the VIS network using tb-fMRI (Fig. 6), permutation analysis showed indications of abnormal network activity masked by network compensation, as evident by the relatively large and similar percentages of significantly decreasing and increasing voxels observed in these networks (Fig. 7). Therefore, this study demonstrates significant changes in clinically relevant functional networks using rs-fMRI and tb-fMRI through ICA, sDL, and permutation analysis approaches in a piglet TBI model.

Children who experience moderate-to-severe TBI often demonstrate functional weakening in brain circuitry and have associated long-term deficits when compared to their peers^34,35^. It has been previously demonstrated that piglets have significant parallels to children in terms of TBI pathophysiology (e.g. intracerebral swelling, white matter damage) and cognitive, behavioral, and motor deficits^8^, making the pig a valuable preclinical model to study the dynamic changes in brain functional networks after TBI. In pediatric TBI patients, damage that occurs to the developing brain often results in abnormal EX network function and long-term functional deficits in cognition, including impaired cognitive flexibility, problem solving, and behavioral control^36–39^. In this study, reduced overall EX network activation was observed using rs-fMRI with ICA and sDL analyses (Table 1 and Fig. 1). Specifically, brain activity reductions were observed in the primary somatosensory, dorsolateral, and anterior prefrontal cortices using sDL^40,41^. In a previous study with an identical CCI TBI piglet model^23^, T-maze testing showed that TBI animals made significantly more wrong choices and took four days longer to make correct choices as compared to control animals. These results suggest reduced problem-solving ability and cognitive flexibility, which support the findings of reduced EX network activity in this study. In a comparable preclinical study, TBI pigs underwent T-maze testing where they learned that a food reward was in a specific arm of the maze^31^. However, once the reward was reversed to the alternate arm, TBI animals spent significantly more time in the old food location attempting to find the reward in comparison to sham pigs, indicating problems in relearning and executive function after insult. TBI pigs have also shown increased fear and anxiety behaviors, including running and escape behaviors in open field testing^42^. Fear and anxiety are modulated by structures associated with the EX network, such as the dorsal anterior cingulate cortex^43^. The results presented here in this work indicate that the entire EX network is significantly impaired, which accurately models what has been shown in pediatric TBI patients^44^ and correlates with previous cognitive and behavioral deficits observed in the piglet TBI model^23^.

Moderate-to-severe TBI in children may also give rise to significant gait deficits, including a reduction in velocity, cadence, and stride length^45–47^, which are correlated with abnormal connectivity throughout the motor networks^48,49^. fMRI studies in humans have demonstrated that the CERE network is also involved in lower limb proprioception and thus gait^50^. The coordination between the SM and CERE networks is important for basic motor tasks, such as limb movement control and processing of touch^51,52^, and TBI leads to aberrant communication between these networks. These gait abnormalities have been characterized in TBI piglets and similarly resulted in decreased velocity, cadence, and stride length^24,25^. In this work, rs-fMRI ICA analysis showed a significant decrease in the premotor cortex activity of the SM network after injury (Table 1 and Fig. 2), which is responsible for motor planning^53^. The premotor cortex is a vital node in the SM network for accurate performance of goal-oriented tasks^54^. This reduction in premotor activity in TBI pigs likely explains previously observed deficits in motor control.

Functional changes in the EX network, known to be associated with the sense of touch and pain^16^, and the SM network, known to be associated with sensory processing, were examined using tactile stimulus and tb-fMRI. When a tactile stimulus was applied, ICA tb-fMRI analysis revealed the overall EX network (including the primary somatosensory, dorsolateral prefrontal, and insular cortices) and the overall SM network (including the primary motor and somatosensory associative cortices) followed an expected decrease in brain activation after injury (Table 1 and Figs. 4, 5). Notably, the primary somatosensory cortex is responsible for receiving tactile information from the body and contributes to the integration of sensory and motor signals for skilled movements that involve other impacted structures, including the primary motor cortex^55^. Thus, an external stimulus, such as a tactile pressure, allows for isolated investigation of individual networks.

Additionally, pigs and humans have similar visual acuity, ability to distinguish colors, and maturational and topographic similarities related to visually evoked responses^56^. The visual cortex in the pig brain is especially similar to the corresponding human visual cortex. Here, in a piglet model of TBI, a significant decrease in the VIS network in TBI animals utilizing rs-fMRI data analyzed by sDL was observed (Table 1 and Fig. 3). Although statistically different Pearson values and mean ratios were not observed for the VIS network using tb-fMRI (Fig. 6), the permutation analysis shows that this network contained relatively high and similar percentages of significantly decreasing and increasing voxels (Fig. 7b). This likely indicates that disruptions in this network were present, and certain areas of the network were compensating for the damaged areas. The decreasing voxels correspond to damaged areas where the TBI group showed reduced activation values in comparison to the control group, and the increasing voxels correspond to the compensating areas where the TBI group showed elevated activation values in comparison to the control group. Situations in which similar percentages of significantly decreasing and increasing voxels are also observed for the SM and CERE networks using rs-fMRI (Fig. 7a) and for the dorsal anterior cingulate cortex of the EX network. Again, this indicates that the areas showing high percentages of increasing voxels within these networks and region are likely compensating for damaged areas (the areas showing high percentages of decreasing voxels).

In this work, the ICA and sDL methods showed consistent patterns, with both detecting disruptions in six of the same individual anatomical structures (the primary visual and associative visual cortices of the VIS network, the primary somatosensory, dorsolateral prefrontal, and anterior prefrontal cortices of the EX network, and the premotor cortex of the SM network) using rs-fMRI and five of the same individual anatomical regions (the primary somatosensory, insular, and ventral anterior cingulate cortices of the EX network and the primary motor and somatosensory associative cortices of the SM network) using tb-fMRI (Table 1). However, despite the consistency in similarly detected disrupted structures by both ICA and sDL, some differences were also observed. Both methods detected disruptions in the insular and ventral anterior cingulate cortices of the EX network using rs-fMRI (Table 1 and Fig. 1); however, ICA detected a significant decrease in connectivity of the TBI group in comparison to the control group, whereas sDL detected a significant increase. Previous work has shown that the ventral anterior cingulate cortex can be effected by anesthesia^9,57^, which may lead to suppressed activations and unreliable measurements in this region. Additionally, some non-significant differing trends between ICA and sDL were also observed within other individual anatomical regions for both rs- and tb-fMRI (regions not denoted by $ in Table 1). However, relatively high consistency was observed across the two methods overall. The tb-fMRI analysis was more consistent with all four networks and 10 out of 13 individual anatomical structures showing similar trends. rs-fMRI analysis was less consistent with only two of the four networks and eight out of 13 individual anatomical regions showing similar trends.

rs-fMRI and tb-fMRI are inherently different. rs-fMRI measures spontaneous synchronized fluctuations in the BOLD signal due to neural firing, whereas tb-fMRI measures fluctuations provoked by performing a specific task or applying a stimulus. Not only are the signal patterns generated by each of these methods different in humans^58^, but recent work suggests that two different neurological processes may govern the underlying mechanisms behind rs- and tb- responses^59^. Other previous works present contradictory results with some suggesting that measured rs- responses are composed of tb- responses and vice versa^15,17–19,60–62^. For example, an earlier study suggested that individual tb- responses are inherent to the individual’s brain and can be predicted from rs- measurements^63^. However, several other human studies yielded evidence that tb- activation of cortical networks shape the pattern of correlated rs- activity^60–62^. It was shown that training for a shape discrimination task induced a negative correlation in the rs- response between the visual cortex and fronto-parietal attentional areas^64^. This study demonstrated that rs- activity may be affected by physiological network level neuro-plasticity arising from experience-driven co-activation of cortical circuitries leading to an anti-correlation between the rs- and tb- responses^65^.

It is still unclear whether or how rs- and tb- functional connectivity relate to each other; therefore, the two different types of functional connectivity may be affected or disrupted differently by TBI, as well as additional factors such as anesthesia. The use of anesthesia is known to interfere with neural activity and neurovascular coupling^57,66,67^. However, several studies have demonstrated that it is still feasible to detect the BOLD response using fMRI in conjunction with isoflurane^68–70^. Vincent et al.^68^ observed similar rs-activations in isoflurane anesthetized monkeys in comparison to tb-activations observed in awake monkeys. This finding supports the perspective that rs-BOLD responses are governed largely by internal dynamics and not derived from consciously directed mental activity^71–75^. It is postulated that RSNs reflect an evolutionary instinct to conserve functional brain organization throughout all levels of consciousness. In this work, minimal anesthesia was used to ensure the pigs were only mildly sedated while remaining immobile. However, individual pigs still may have responded differently to the anesthesia than others due to biological factors, and anesthesia may have affected certain anatomies more than others. Therefore, confounding factors, including differing neurological processes governing rs- and tb- responses and possible neural suppression from anesthesia, may have had an effect on the observed differences between the rs- and tb- results.

The differing neurological processes governing rs- and tb- responses may also explain the differences in sensitivity of ICA and sDL to the two types of fMRI observed in this study. sDL appeared to be more sensitive to changes in rs- activity between the TBI and control groups, as observed by lower p-values and more individual anatomical structures showing significant differences (Table 1), while ICA seemed to be more sensitive to changes in tb- activity. Recent work also showed that sDL was superior to ICA in detecting healthy RSNs using a pig model^9^. sDL is different from ICA in that it does not assume independent signal components. It allows for the possibility of the signal to contain independent and/or dependent components. As discussed above, it is still unclear whether or how rs- and tb- functional connectivity relate to each other. Therefore, using an independent (ICA) or dependent-allowing (sDL) signal decomposition may be more beneficial depending on whether rs- or tb- data is being evaluated.

One limitation of this study is the small group sizes (n ≤ 7). In the reference atlas analysis (Table 1 and Figs. 1, 2, 3, 4, 5, 6), it is possible that Type II errors (false negatives) may have occurred, meaning significant differences between the control and TBI groups may have existed for certain networks and/or individual anatomies but were unable to be detected due to insufficient statistical power. It is also possible that Type I errors (false positives) of up to 5% may have occurred in the permutation analysis (Figs. 7–8). In this work, no correction for multiple comparisons was employed due to the relatively small number of possible permutations (≤ 792), which dictates the lowest achievable p-value (one divided by the number of permutations). Multiple comparison correction techniques^76–78^ were found to be too conservative, providing corrected significance levels that were too small to detect given the small number of possible permutations and number of voxels being evaluated (≥ 2400). However, even though the two types of analyses performed in this study may be susceptible to the two different types of errors, consistent results were observed, suggesting that the observed functional connectivity disruptions are legitimate. Another limitation of this study is the lack of a sham control group to account for the pre-operative antibiotics and post-operative medications, since the healthy control pigs did not receive these medications. Future studies should include a sham animal group to account for these important variables.

In conclusion, this work successfully demonstrates for the first time in a pediatric piglet TBI model that rs- and tb-fMRI can detect functional connectivity disruptions caused by TBI with relatively high consistency in activation trends utilizing ICA and sDL analysis approaches. Significant differences (p < 0.05) were observed in the VIS, EX, and SM networks between the control and TBI groups, as well as within several individual anatomical structures. Functional disruptions spanning the entire brain, well beyond the primary lesioned cerebral cortex, in TBI pigs were observed, which agrees with clinical studies that found moderate-to-severe TBIs result in whole brain functional connectivity alterations and diminished network dynamics^79,80^. This study opens the door for future research to test the effects of novel therapies that alter brain network connectivity and improve recovery in the piglet TBI model; and thus, hasten the translation of treatments to the clinical patient population.

## Materials and methods

### Subjects

Four-week-old, castrated male and intact female Landrace-cross piglets (n = 12) were used in this study. These juvenile animals were pre-pubescent, and therefore, differences in reproductive hormones and comparable post-pubertal differences were assumed to be minimal. TBI was induced in five (n = 5; n = 2 male, n = 3 female) pigs using the previously published procedure^25^, while the remaining seven (n = 7; n = 4 male, n = 3 female) served as controls (normal, healthy pigs). All experimental procedures were approved by the Institutional Animal Use and Care Committee (IAUCC, University of Georgia; Protocol Number A2019 07-007-Y1) and performed in accordance with the National Institutes of Health (NIH) Guidelines for the Care and Use of Laboratory Animals. All animals were included in the study and the experiments have been reported following/in compliance with the Animal Research: Reporting in Vivo Experiments (ARRIVE) guidelines. Pigs were group housed in a Public Health Service (PHS) and Association for Assessment and Accreditation of Laboratory Animal Care (AAALAC) approved facility at room temperature (approximately 27 °C) with a 12-h light/dark cycle. Pigs encountered daily enrichment through toys and human contact and were fed standard pig starter 1 diets.

### Controlled cortical impact

Pigs were anesthetized, and a periosteal block (0.5% bupivacaine; 2 mg/kg; Pfizer) at the cranium was applied under aseptic technique. A craniectomy was performed, approximately 20 mm in diameter, to expose the underlying dura at the left anterior junction of the coronal and sagittal sutures. Each pig was secured in a controlled cortical impactor device (University of Georgia Instrument Design and Fabrication Shop, Athens, GA), and a 15 mm impactor tip was positioned over the intact dura to induce injury with the following parameters: velocity of 4 m/s, depth of depression of 9 mm, and dwell time of 400 ms. These parameters were based on previous studies to generate a moderate TBI^25,81,82^.

Pre-operatively, TBI pigs received antibiotics (ceftiofur crystalline free acid; 5 mg/kg intramuscular (IM); Zoetis). Pre-induction sedation and analgesia for TBI surgery was achieved using buprenorphine (0.01 mg/kg IM; Covetrus), midazolam (0.2 mg/kg IM; Heritage), and xylazine (2 mg/kg IM; VetOne). Prophylactic lidocaine (0.5 mL 2% lidocaine; VetOne) was topically applied to laryngeal folds, and propofol (to effect, intravenous (IV); Zoetis) was administered to achieve intubation. Anesthesia was maintained with isoflurane (2.0%; Abbott Laboratories) in oxygen. Post-operatively, once vitals returned to normal ranges, the pigs were monitored every four hours for 24 h and then twice a day. For pain and inflammation maintenance, pigs received buprenorphine (0.01 mg/kg IM; Covetrus) every eight hours for 24 h and banamine (2.2 mg/kg IM; Merck) every 12 h for 24 h, and then every 24 h for 72 h post-operatively. TBI pigs displayed contralateral ataxia. Healthy control pigs did not receive pre-operative antibiotics or post-operative medications.

### Data acquisition

One day post-TBI, pigs were initially sedated using xylazine (2 mg/kg IM; VetOne), ketamine (4 mg/kg IM; Henry Schein), and midazolam (0.2 mg/kg IM; Heritage). Then mild anesthesia was maintained with inhalational isoflurane (1.5%; Abbott Laboratories) in oxygen in order to keep the pigs sedated while reducing anesthetic agent interference with pig brain neural activity and neurovascular coupling.

Using a GE 32-channel fixed-site Discovery MR750 3.0 Tesla MRI magnet and 8-channel knee coil, T1-weighted anatomical, rs-fMRI, and tb-fMRI data was acquired using the following two sequences: (1) 3D fast spoiled gradient echo (FSPGR) sequence (repetition time TR = 5.5 s, echo time TE = 2.1 ms, flip angle FA = 9°, field-of-view FOV = 12.8 × 12.8x6.4 cm, slice thickness = 1 mm, a reconstruction matrix size of 256 × 256x112 (resulting in cubic voxels of 0.5 mm), axial slice plane, and an acquisition time of 10 min 57 s), and (2) gradient echo-planar imaging (EPI) sequence (TR = 3 s, TE = 30 ms, FA = 80°, FOV = 12.8 × 12.8x6.2 cm, a matrix size of 96 × 96x31, coronal slice plane, 305 total volumes for rs-fMRI (an acquisition time of 15 min 15 s) and 125 volumes for tb-fMRI (6 min 15 s)).

The tb-fMRI data was acquired in a block design paradigm with the first 15 s being dead time (stimulus off) to allow the magnetization to reach a steady state, followed by six repetitions of 30 s stimulus ON and 30 s stimulus OFF cycles. The visual stimuli were generated bilaterally using a high-intensity 300 W xenon arc lamp (Cermax, Excelitas Technologies, Fermont, CA) and a specially made high-fidelity fiber optic bifurcated cable (Atlas Specialty Lighting). This lamp produced a color rendering index (CRI) of 98, which demonstrated similarity to noon-day sunlight (CRI = 100). The pig was in supine position, and the fiber optic cable was positioned to direct light into both the pig’s left and right closed eyes. The tactile stimuli were also generated bilaterally by placing MRI-safe plastic hemostats with locking mechanisms on the inguinal region to activate the saphenous nerve that innervates the skin of the inner hind limbs^83^.

The rs-fMRI data was acquired for all twelve pigs (n = 7 controls and n = 5 TBIs), while tb-fMRI data with visual stimuli was acquired for eleven pigs (n = 6 controls and n = 5 TBIs) and tb-fMRI data with tactile stimuli was acquired for nine pigs (n = 4 controls and n = 5 TBIs).

### Data preprocessing

Each fMRI dataset was preprocessed to realign images to correct for motion and perform slice-timing correction using Statistical Parametric Mapping (SPM) software^84^. For motion correction, all acquired rs-fMRI and tb-fMRI volumes for each pig were aligned to that pig’s corresponding first acquired rs-fMRI volume. All fMRI datasets and T1-weighted anatomical images were skull stripped by manual slice-by-slice segmentation. The first five volumes of each fMRI dataset were removed to allow the magnetization to reach a steady state; therefore, 300 total volumes were remaining for rs-fMRI data and 120 for tb-fMRI data.

To determine spatial activation maps for each individual pig (***α***_***i***_), functional connectivity analysis was performed using two methods: (1) group independent component analysis (ICA) with back reconstruction and (2) group sparse dictionary learning (sDL) with dual regression. First, group datasets were created and group activation maps were generated using a previously published procedure^9^. In summary, one pig from the control group and one from the TBI group was selected as the template pig based on visual inspection of the fMRI data taking into consideration distortion, artifacts, and the signal-to-noise ratio. Then the remaining pigs within each group were spatially normalized to the template pig using the first volume of each rs-fMRI dataset to calculate a transformation. The calculated transformation for each pig was then applied to the rest of the prior motion-corrected volumes (including both rs-fMRI and tb-fMRI volumes) of that corresponding pig. Spatial normalization was accomplished using SPM’s Old Normalize algorithm^84^. For the control group and TBI group, separate group datasets for rs-fMRI, tb-fMRI with visual stimuli, and tb-fMRI with tactile stimuli were created by temporally concatenating the corresponding fMRI time series of the spatially normalized pigs at each voxel.

Therefore, a total of six group datasets were created: a rs-fMRI control group dataset with 2100 volumes (7 pigs with 300 volumes each), a rs-fMRI TBI group dataset with 1500 volumes (5 pigs with 300 volumes each), a tb-fMRI with visual stimuli control group dataset with 720 volumes (6 pigs with 120 volumes each), a tb-fMRI with visual stimuli TBI group dataset with 600 volumes (5 pigs with 120 volumes each), a tb-fMRI with tactile stimuli control group dataset with 480 volumes (4 pigs with 120 volumes each), and a tb-fMRI with tactile stimuli TBI group dataset with 600 volumes (5 pigs with 120 volumes each).

### Data analysis

Group ICA with back reconstruction and group sDL with dual regression were performed on each group dataset to generate spatial activation maps for each individual pig (***α***_***i***_).

Spatial group ICA was performed using the Group ICA of fMRI Toolbox’s (GIFT) Infomax algorithm^85^, which includes dimensionality reduction using principle component analysis (PCA), to produce group activation maps (***α***_***g***_). Back reconstruction was then performed using ***α***_***g***_ and the results from the data reduction step to obtain ***α***_***i***_ for each individual pig^86^. Both the group and individual datasets were decomposed into 70 independent components, which is a value commonly used throughout the literature.

sDL was performed using the SPArse Modeling Software (SPAMS) toolbox^87^ to minimize the equation

1 $$\underset{{\varvec{D}}\in C}{\mathrm{min}}\underset{n\to +\infty }{\mathrm{lim}}\frac{1}{n}\sum_{j=1}^{n}\left(\underset{{\alpha }^{j}}{\mathrm{min}}{\frac{1}{2}{\Vert {{\varvec{s}}}^{j}-D{\boldsymbol{\alpha }}^{j}\Vert }_{2}}^{2}+\lambda {\Vert {\boldsymbol{\alpha }}^{j}\Vert }_{1}\right)$$

where ***s*** is the fMRI dataset, ***D*** is the dictionary or associated time series, *λ* is a sparsity parameter, and *n* is the number of atoms or dictionary elements the fMRI data is decomposed into.

When performing the sDL analysis, ***α***_***g***_ was first obtained by minimizing Eq. (1) for each group dataset, and dual regression was then performed to obtain ***α***_***i***_ for each individual pig. The first regression used ***α***_***g***_ and each pig’s individual rs-fMRI data (**s**_**i**_) to determine an individual dictionary (***D***_***i***_) or associated time series using linear least squares to solve the equation **s**_**i**_ = ***D***_***i***_*** α***_***g***_. The second regression then used each pig’s ***D***_***i***_ and **s**_**i**_ to determine ***α***_***i***_ by minimizing Eq. (1).

Note that the difference between back reconstruction and dual regression is that back reconstruction uses the group result (***α***_***g***_) and the results of the data reduction step to generate ***α***_***i***_, whereas dual regression uses the group result (***α***_***g***_) and the original fMRI data (**s**_**i**_)^88^. When performing sDL, it is not common to perform dimensionality reduction, and the use of a complete or over-complete dictionary is often employed to achieve sparseness^87^. For this study, dictionaries with *n* = 300 and *n* = 120 were used for the rs-fMRI and tb-fMRI datasets, respectively, as these values correspond to the time series length (number of volumes) of the acquired fMRI datasets. A sparsity parameter *λ* = 0.15 was also used when solving for all individual and group activation maps, which was based on a previous optimization procedure^9^.

All individual pig activation maps were normalized by the maximum activation value from the corresponding group dataset, thresholded using a Z-score of one, and then spatially normalized to the template pig’s anatomical space. Spatial normalization was accomplished using SPM to calculate a spatial transformation between the template pig’s first rs-fMRI volume and its corresponding T1-weighted anatomical data. The transformation was then applied to the activation maps for all pigs within the template pig’s group. When applying these transformations, voxel size was reshaped to 1 mm cubed, opposed to the 0.5 mm cubed voxels of anatomical space, in order to reduce the computational size of the activation map datasets.

### Pig reference functional connectivity atlases and activation map analysis

Next, reference pig functional connectivity atlases were created also using the same previously published procedure^9^. First, a standard pig brain atlas^33^ was spatially normalized to the T1-weighted anatomical space of each template pig. Spatial normalization was accomplished by calculating a spatial transformation using T1-weighted anatomical data provided in the same space as, and associated with, the standard pig brain atlas and the T1-weighted anatomical data acquired for each template pig. The calculated transformation was then applied to the atlas, and when applying this transformation, voxel size was again reshaped to 1 mm cubed. Using the anatomical structures provided in Table 1 and Table S1 of the Supplementary Material, seven reference functional connectivity atlases were created for each pig: visual (VIS), executive control (EX), sensorimotor (SM), cerebellar (CERE), default mode (DMN), salience (SAL), and basal ganglia (BAS) networks.

With the activation maps and reference atlases in the same space, Pearson spatial correlation coefficients and mean ratios (defined as mean activation value within a given atlas divided by the mean activation value outside of the given atlas) were calculated for each activation map and reference atlas. The same reference atlases were used for both rs- and tb-fMRI analysis, as the rs- and tb-networks were assumed to be comprised of identical anatomies. For activation maps generated using the ICA analysis, the map for each fMRI dataset and reference atlas that produced the largest Pearson value was determined. From these maximal maps, Pearson values and mean ratios were then calculated for each individual anatomical structure comprising each corresponding reference atlas, as listed in Tables 1 and S1 under each network. For the CERE atlas, the individual lobes were not examined. Instead, the CERE network is discussed as a whole in conjunction with the SM network, as it has been shown that coordination between these networks is necessary for basic motor tasks to occur^50^.

Since sDL generates multiple similar activation maps with near maximum Pearson values, the three maps (corresponding to three separate atoms in the learned dictionary) for each fMRI dataset and reference atlas that produced the largest Pearson values were determined and averaged to produce final activation maps. Pearson values and mean ratios were then re-calculated for these final maps and each corresponding reference atlas, as well as for each individual anatomical structure comprising each corresponding reference atlas.

P-values associated with the Pearson values and mean ratios were calculated between the TBI group and the control group for each network as a whole, as well as for each individual anatomical region, using a two-sample t-test assuming the groups had unequal variances. For each metric, the groups were considered to be significantly different if p < 0.05, and a network or anatomical structure was only considered significantly different if the p-values for both the Pearson values and mean ratios were below 0.05.

### Permutation analysis

To locate significantly different voxels between the TBI and control groups, permutation tests were performed using the activation maps generated by the sDL analysis^77^. Pigs in the TBI group were registered to the control group using SPM’s Old Normalize algorithm^84^. Spatial normalization was accomplished by calculating a spatial transformation between the T1-weighted anatomical datasets of the template pigs for the TBI and control groups. The transformation was then applied to the activation maps for all pigs within the TBI group, which had previously been spatially normalized to the TBI template pig’s T1-weighted anatomical space (“Data analysis” section).

For permutation tests, the null hypothesis stated that no statistical difference existed between the mean activation values of the TBI and control groups^77^. For ${N}_{C}$ pigs in the control group and ${N}_{T}$ pigs in TBI group, there were ${C}_{{N}_{C}+{N}_{T}}^{{N}_{C}}$ possible combinations of arranging all pigs into the two groups. For every possible combination, the difference in the mean activation values of the two groups

2 $$T=mea{n}_{C}-mea{n}_{T}$$

were calculated on a voxel-by-voxel basis. For each voxel, if the true experimental combination of control and TBI groups created a mean difference ${(T}_{actual})$ among the largest five percent of all calculated *T* values (α-level of 0.05), the null hypothesis was rejected, and the activation values of the TBI group were considered to be significantly decreased in comparison to the control group. If ${T}_{actual}$ was among the lowest five percent (negative number), then the activation values of the TBI group were considered to be significantly increased in comparison to the control group. Permutation tests were performed for every voxel throughout the brain, and the percentage of significantly decreasing and increasing voxels within each network’s corresponding reference atlas and comprising individual anatomical structures were calculated.

## Supplementary Information

Supplementary Information.

## Data Availability

Data and MatLab code will be available upon reasonable request.
