# Supplementary material for: Detecting functional connectivity disruptions in a translational pediatric traumatic brain injury porcine model using resting-state and task-based fMRI
Source: Sci Rep. 2021 Jun 11;11:12406. doi: 10.1038/s41598-021-91853-5 (PMC8196021; doi:10.1038/s41598-021-91853-5)
Supplement: Supplementary file 1 — Supplementary Information. [file 41598_2021_91853_MOESM1_ESM.pdf]

Detecting functional connectivity disruptions in a translational pediatric traumatic brain injury porcine model using resting-state and task-based fMRI

Gregory Simchick[1,2]#, Kelly M. Scheulin[2,3,4]#, Wenwu Sun[1,2], Sydney E. Sneed[2,4], Madison M. Fagan[2,3,4], Savannah R. Cheek[4], Franklin D. West[2,3,4]*, Qun Zhao[1,2]*

[1] University of Georgia, Franklin College of Arts and Sciences, Department of Physics and Astronomy, Athens, GA, USA

[2] University of Georgia, Regenerative Bioscience Center, Athens, GA, USA

[3] University of Georgia, Biomedical and Health Sciences Institute, Neuroscience Program, Athens, GA, USA

[4] University of Georgia, College of Agricultural and Environmental Sciences, Department of Animal and Dairy Science, Athens, GA, USA

# These authors contributed equally to this work and are considered to be co-first authors.

* Corresponding authors: [425 River Road Rm 316; Athens, GA 30602; T: 706-542-0988 F: 706-542-7925; westf@uga.edu] (Franklin D. West) and [500 D.W. Brooks Drive Rm 119; Athens, GA 30602; T: 706-583-5558; qunzhao@uga.edu] (Qun Zhao)

Supplementary Material

S3. Supplementary Tables and Figures

Table S1: P-Values Comparing Control vs TBI Group

rs-fMRI Networks and Anatomical Regions	ICA Sig	ICA Pearson	ICA Mean Ratio	sDL Sig	sDL Pearson	sDL Mean Ratio
**Default Mode**[$]	-	0.95	0.99	-	0.55	0.98
Hippocampus[$]	-	0.08	0.11	-	0.10	0.36
Medial Anterior Prefrontal Cortex[$]	-	0.14	0.13	-	0.45	0.46
Orbitofrontal Cortex[$]	-	0.43	0.44	-	0.29	0.29
Inferior Temporal Gyrus	*	<0.01*	<0.01*	[#]	<0.01[#]	<0.01[#]
Ventral Posterior Cingulate Cortex	-	0.06	0.07	*	0.01*	<0.01*
Dorsal Posterior Cingulate Cortex	-	0.54	0.68	-	0.10	0.12
Retrosplenial Cingular Cortex	-	0.36	0.35	*	0.04*	0.04*
Anterior Entorhinal Cortex	-	0.70	0.55	-	0.24	0.64
Parahippocampal Cortex	*	0.02*	0.01*	-	0.66	0.56
**Salience**[$]	-	0.18	0.54	-	0.35	0.41
Caudate Nucleus	[#]	0.01[#]	0.01[#]	-	0.08	0.21
Globus Pallidus	[#]	0.02[#]	0.04[#]	-	0.58	0.62
Insular Cortex	-	0.97	0.49	-	0.21	0.45
Middle Temporal Gyrus	-	0.04*	0.10	-	0.70	0.34
Ventral Posterior Cingulate Cortex[$]	-	0.61	0.62	-	0.59	0.98
Ventral Anterior Cingulate Cortex[$]	-	0.36	0.39	-	0.27	0.37
Retrosplenial Cingular Cortex[$]	-	0.15	0.17	-	0.12	0.53
Dorsal Posterior Cingulate Cortex	[#]	0.04[#]	0.04[#]	-	0.91	0.82
Dorsal Anterior Cingulate Cortex	*	<0.01*	<0.01*	-	0.21	0.20
**Basal Ganglia**	-	0.33	0.24	-	0.03[#]	0.06
Thalamus	*	<0.01*	<0.01*	[#]	<0.01[#]	<0.01[#]
Caudate Nucleus	*	0.01*	0.02*	-	0.99	0.87
Putamen	-	0.99	0.96	-	0.14	0.15
Amygdala	-	0.78	0.95	-	0.11	0.13
Primary Motor Cortex[$]	-	0.75	0.81	-	0.58	0.66
Orbitofrontal Cortex	-	0.49	0.48	-	0.68	0.68
Parahippocampal Cortex[$]	-	0.32	0.27	*	<0.01*	<0.01*

P-values comparing the Pearson spatial correlation coefficients and mean ratios obtained from the resting-state (rs-) fMRI analysis for the control group and the TBI group for each network and each individual anatomical region. For each metric, the groups were considered significantly different if $p<0.05$ (denoted by * for a significant decrease in the TBI group in comparison to the control group and [#] for a significant increase). However, a network or anatomical region was only considered significantly different if the p-values for both the Pearson values and mean ratios were below 0.05 ('Sig' columns). Networks and regions that showed consistent trends across ICA and sDL are denoted by [$].

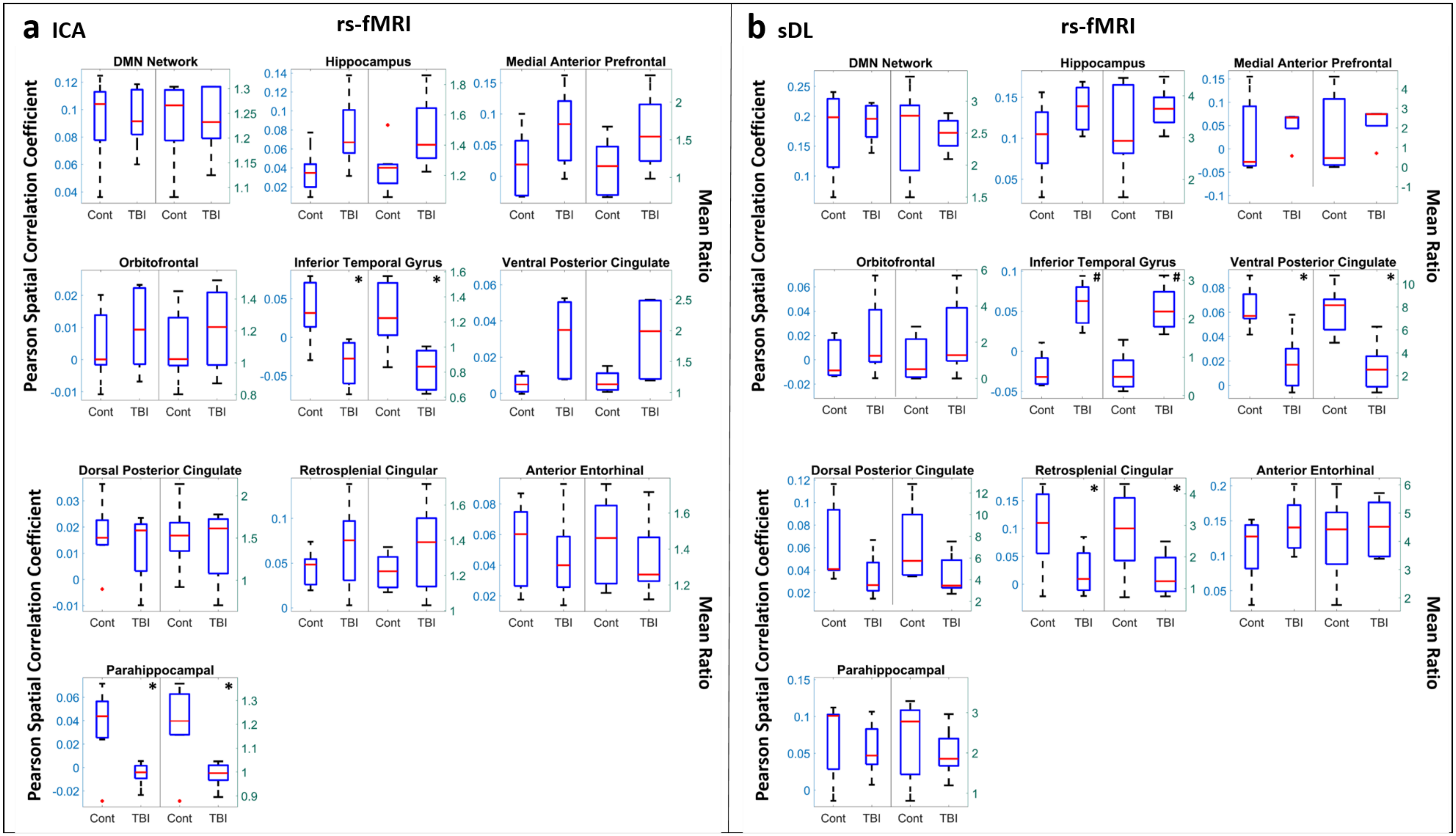

Figure S1: Boxplots of Pearson spatial correlation coefficients (left) and mean ratios (right) obtained from the resting-state (rs-) analysis for the default mode (DMN) network and its individual anatomical regions obtained using independent component analysis (ICA; **a**) and sparse dictionary learning (sDL; **b**). The red line denotes the median, the blue box denotes the interquartile range, and the lower and upper whiskers denote the min and max, respectively. An asterisk (*) or pound (#) in the upper right-hand corner indicates significant decrease or increase ($p<0.05$), respectively, for the TBI group in comparison to the control group.

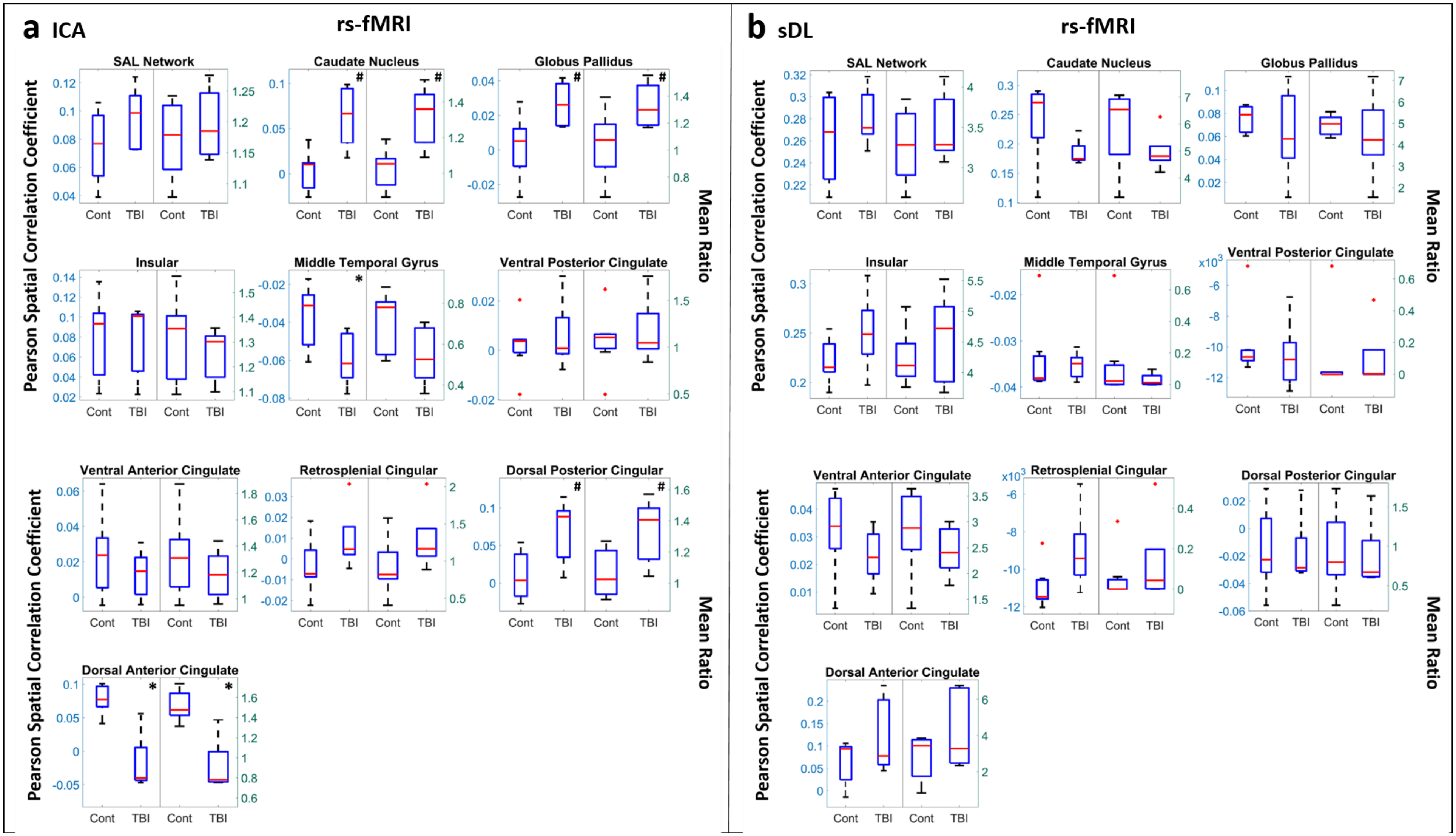

Figure S2: Boxplots of Pearson spatial correlation coefficients (left) and mean ratios (right) obtained from the resting-state (rs-) analysis for the salience (SAL) network and its individual anatomical regions obtained using independent component analysis (ICA; **a**) and sparse dictionary learning (sDL; **b**). The red line denotes the median, the blue box denotes the interquartile range, and the lower and upper whiskers denote the min and max, respectively. An asterisk (*) or pound ($^{\#}$) in the upper right-hand corner indicates significant decrease or increase ($p<0.05$), respectively, for the TBI group in comparison to the control group.

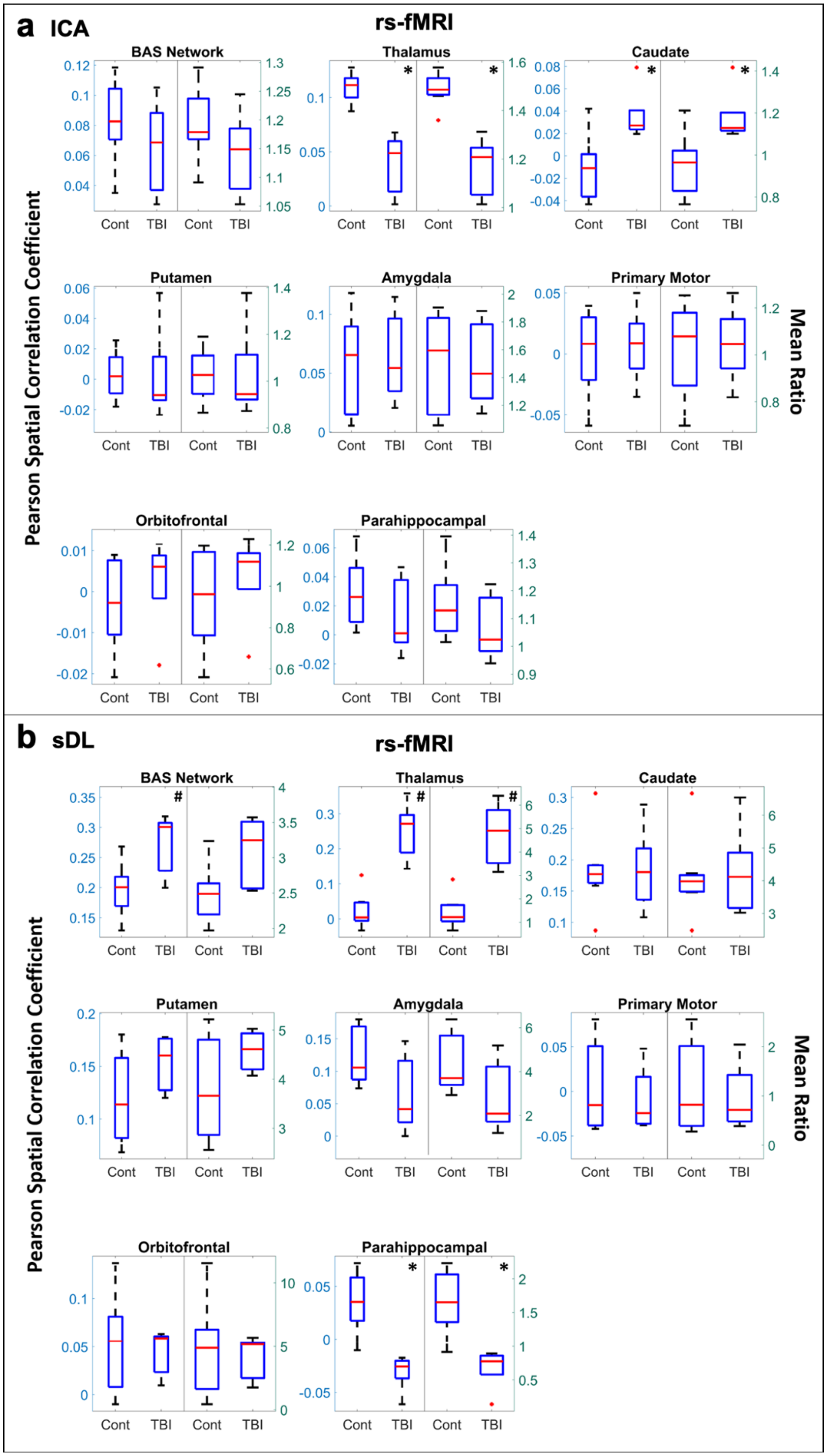

Figure S3: Boxplots of Pearson spatial correlation coefficients (left) and mean ratios (right) obtained from the resting-state (rs-) analysis for the basal ganglia (BAS) network and its individual anatomical regions obtained using independent component analysis (ICA; **a**) and sparse dictionary learning (sDL; **b**). The red line denotes the median, the blue box denotes the interquartile range, and the lower and upper whiskers denote the min and max, respectively. An asterisk (*) or pound ($^{\#}$) in the upper right-hand corner indicates significant decrease or increase ($p<0.05$), respectively, for the TBI group in comparison to the control group.

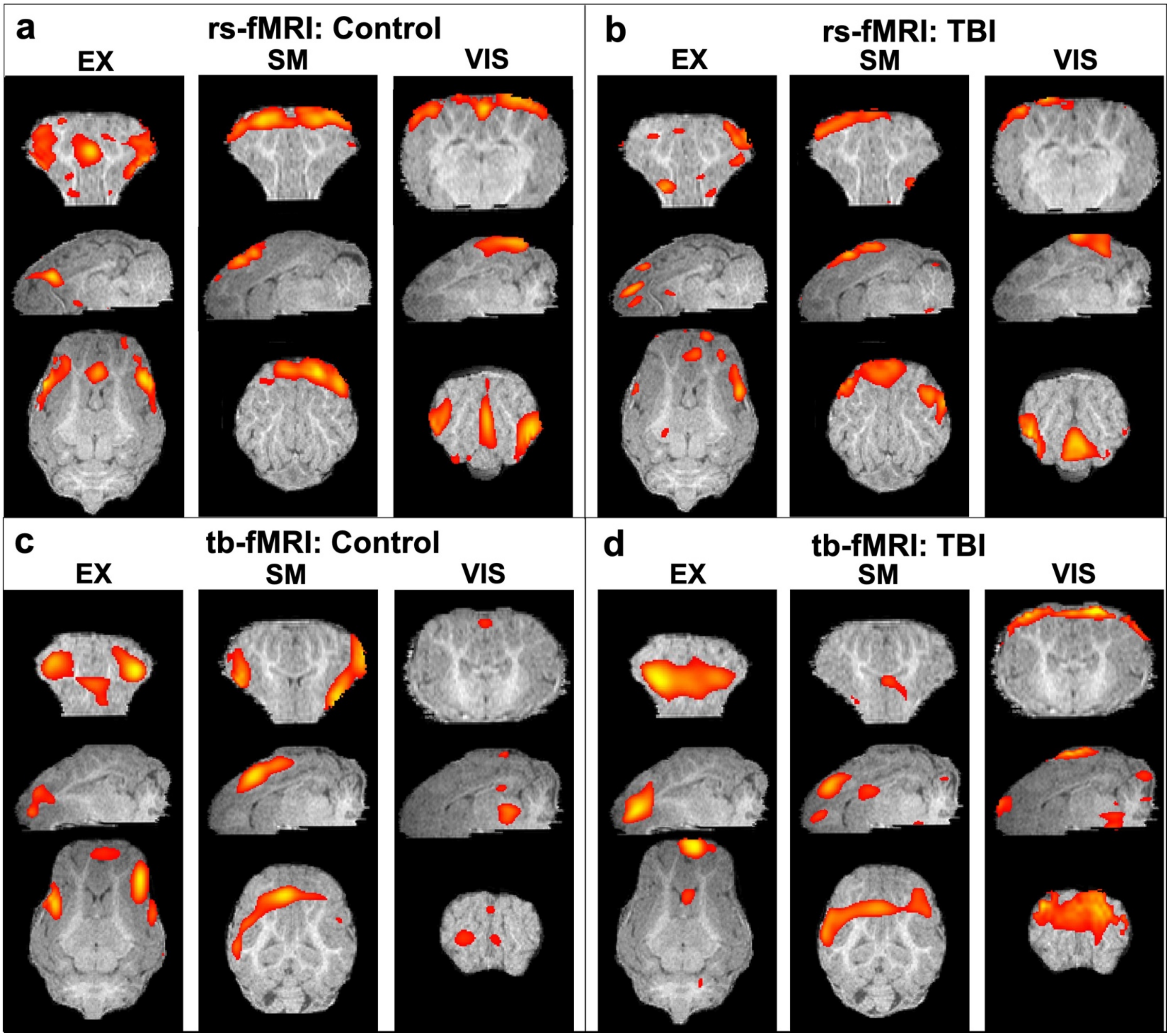

Figure S4: Representative images of two-dimensional activation maps from the resting-state (rs-; a and b) and task-based (tb-; c and d) ICA fMRI analysis for the control (a and c) and TBI (b and d) groups for the executive control (EX), sensorimotor (SM), and visual (VIS) networks overlaid on the template pigs' T1-weighted anatomical images.

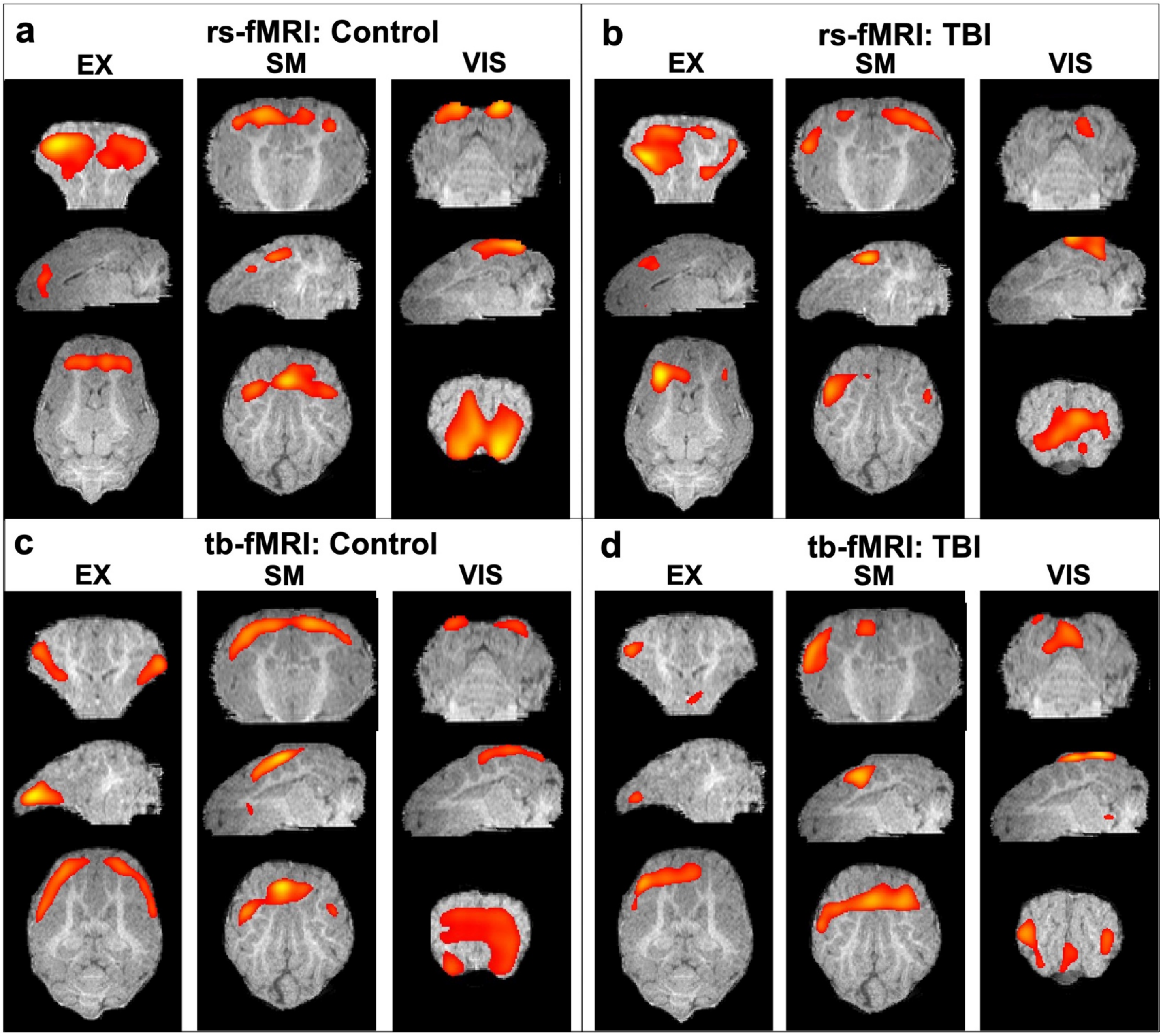

Figure S5: Representative images of two-dimensional activation maps from the resting-state (rs-; a and b) and task-based (tb-; c and d) sDL fMRI analysis for the control (a and c) and TBI (b and d) groups for the executive control (EX), sensorimotor (SM), and visual (VIS) networks overlaid on the template pigs' T1-weighted anatomical images.
